# Mechanism driven adaptation of smart hydrogels to the osteoarthritis pathological microenvironment

**DOI:** 10.3389/fbioe.2026.1751293

**Published:** 2026-03-12

**Authors:** Haoming You, Qiuyuan Liu, Jingyi Zhang, Pai Zheng, Wenbo Dou, Yichen Yao, Junjie Li, Fangyue Wang, Yong Huang

**Affiliations:** 1 Department of Orthopedics, Hospital of Chengdu University of Traditional Chinese Medicine, Chengdu, China; 2 Institute of Traditional Chinese Medicine, Sichuan Academy of Chinese Medicine Sciences, Chengdu, China; 3 Chongqing University of Traditional Chinese Medicine Bone Injury Teaching and Research Office, Chong Qing, China

**Keywords:** drug delivery, osteoarthritis treatment, pathogenesis mechanisms, smart hydrogels, tissue engineering

## Abstract

Osteoarthritis (OA) arises from interconnected pathological processes, including persistent inflammation, mitochondrial dysfunction, cartilage matrix degeneration, and abnormal neurovascular remodeling. Current clinical care remains largely symptomatic and targets only a narrow set of mechanisms, which limits modification of the disease course. Smart hydrogels, owing to their injectability, biocompatibility, and responsiveness to intrinsic and extrinsic cues, offer notable advantages for OA therapy. By sensing changes in the joint microenvironment, they enable precise control of drug release in space and time and shift treatment from symptomatic control toward targeted repair. This review first synthesizes the roles and interactions of the principal mechanisms that shape the OA microenvironment. It then surveys recent advances in smart hydrogels for OA, with emphasis on applications that suppress inflammation, regulate mitochondrial function, promote cartilage repair, and modulate abnormal neurovascular remodeling. Design strategies for responsive crosslinking networks and their integration with delivery vehicles such as bioactive molecules, nanomaterials, and exosomes are also outlined. Remaining challenges are discussed, including harmonized efficacy endpoints, durability and safety *in vivo*, scalable manufacturing, and translation to clinical practice, together with opportunities for future research. By coupling mechanistic insight with materials design, this review highlights the potential of smart hydrogels to deliver microenvironment adaptive, multitarget interventions and aims to support rational optimization of new materials and progress toward clinical.

## Graphical Abstract

**Figure:**
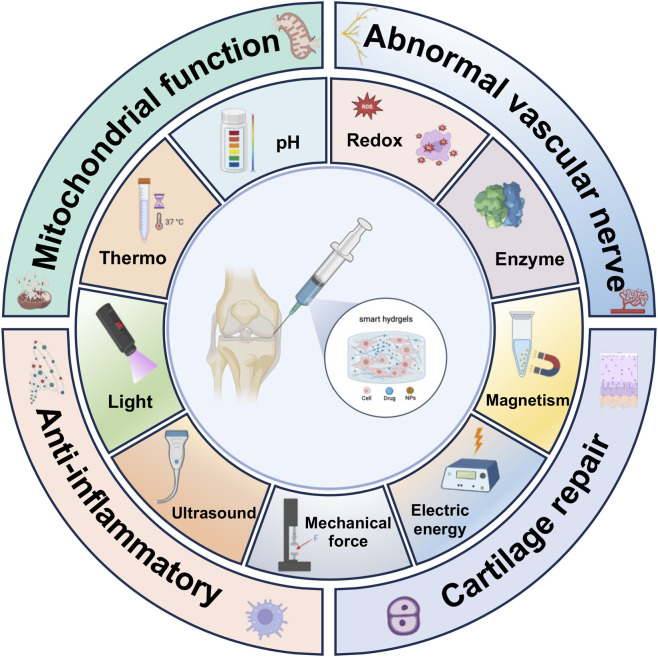
Schematic of Smart Hydrogel Targeting Pathogenic Mechanisms to Treat Osteoarthritis.

## 1 Introduction

Osteoarthritis (OA) is a complex degenerative joint disease and a leading cause of disability among older adults, imposing a substantial socioeconomic burden on individuals, families, and society. Globally, an estimated 500 to 600 million people—about 7% of the population—are affected. The risk of OA rises markedly with advancing age as joint structures deteriorate, and it is further amplified by prolonged exposure to risk factors such as prior joint injury and obesity (Steinmetz et al., 2023; Zhou R. et al., 2024; Tang et al., 2025). Clinically, pain, deformity, and functional limitation predominate and markedly reduce quality of life (Binvignat et al., 2024). However, current treatments remain limited. Despite the urgent need for effective therapies, no approved option reliably reverses or even slows the progressive structural degeneration characteristic of OA (Duong et al., 2023). Consequently, research increasingly targets key pathological processes, including articular cartilage degeneration, remodeling of the subchondral bone, osteophyte formation, and synovitis (Abramoff and Caldera, 2020; Tang et al., 2025).

The pathogenesis of osteoarthritis reflects coordinated effects of genetic, metabolic, biomechanical, and immune factors, which together confer substantial complexity (Zheng et al., 2019; Boer et al., 2021; Wood et al., 2022). Early alterations arise primarily in the synovium, where lining hyperplasia and fibrosis are observed. In parallel, proinflammatory cytokines become upregulated in chondrocytes, osteoblasts, and synovial fibroblasts, activating mitogen-activated protein kinase (MAPK) and nuclear factor κB (NF-κB) signaling. Notably, interleukin-1β binds the IL-1 receptor type I and induces enzymes that degrade the extracellular matrix, including matrix metalloproteinases (MMPs) and a disintegrin and metalloproteinase with thrombospondin motifs (ADAMTS), thereby amplifying synovitis and initiating progressive cartilage loss (Li et al., 2021; Liew et al., 2023; Wang et al., 2024). At the same time, these mediators drive heightened mitochondrial respiration in chondrocytes, generating excess reactive oxygen species (ROS) that damage mitochondria and promote chondrocyte apoptosis and autophagy (Vonk et al., 2018; Li et al., 2025a). Because early symptoms are insidious, inflammation within the synovium and joint space often intensifies by the intermediate stage, and lesions extend into the cartilage, with extracellular matrix (ECM) degeneration, fibrosis within calcified cartilage, and chondrocyte hypertrophy (Mahmoudian et al., 2021). Elevated cytokines and activation of NF-κB, MAPK, and Wnt pathways sustain the release of multiple MMPs, disrupt oxidative phosphorylation, perturb mitochondrial membrane potential and cellular energetics, and thereby exacerbate cartilage damage (Jin et al., 2022; Li et al., 2022). In advanced disease, pathology involves the entire joint and presents as abnormal bone remodeling and sclerosis. Chronic oxidative stress promotes mutations in mitochondrial DNA (mtDNA) and depletion of ATP, culminating in extensive chondrocyte apoptosis. Concurrently, transforming growth factor beta (TGF-β) and vascular endothelial growth factor (VEGF) signaling in the cartilage microenvironment accelerates vascular invasion; by stimulating mesenchymal stem cells (MSCs), angiogenesis is enhanced and neovessels penetrate the calcified cartilage into the articular cartilage, driving chondrocyte proliferation and ectopic bone formation (Rim et al., 2020; Hu Y. et al., 2021; Mendelsohn et al., 2025). In addition, nerve growth factor (NGF) activates TrkA receptors, fostering ingrowth of sensory fibers at the osteochondral junction and heightening pain sensitivity, thereby establishing a chronic pain state (Zhao L. et al., 2024). Collectively, inflammatory signaling, mitochondrial dysfunction, aberrant neurovascular invasion, and cartilage destruction act in concert to propel OA progression.

Traditional management of osteoarthritis (OA) aims to relieve symptoms and improve function and can be divided into nonsurgical and surgical approaches. Nonsurgical care includes lifestyle modification, physical and exercise therapy, oral or intravenous nonsteroidal anti-inflammatory drugs (NSAIDs) and opioids, and intraarticular injections of corticosteroids, hyaluronic acid (HA), and other agents (Duong et al., 2023). Although these therapies may modestly slow progression, they offer limited capacity for cartilage repair or regeneration, often have low intraarticular bioavailability, and frequently cause gastrointestinal, renal, and cardiovascular adverse effects (Ma L. et al., 2022). Stem-cell therapy, platelet-rich plasma, and newer targeted drugs have been proposed, but they are not yet widely recommended because supporting evidence remains insufficient (Hochberg et al., 2019; Paget et al., 2021; Sadeghirad et al., 2024). Advanced OA often necessitates arthroscopic debridement, osteotomy, or joint replacement; however, these procedures are associated with complications and substantial perioperative burden (Pacheco-Brousseau et al., 2024; Cotter et al., 2025). The limitations of current care highlight an urgent need for new therapeutic strategies. Hydrogels are hydrophilic 3D polymer networks composed of water and a crosslinked matrix. They exhibit excellent biocompatibility, degradability, and tunable mechanics, and they can provide a permissive cellular microenvironment (Xv et al., 2025). Because they resemble the native extracellular matrix (ECM), hydrogels are well suited to carry bioactive molecules or cells and to deliver them to the joint (Choi et al., 2021; Duan et al., 2023). Over the past 2 decades, applications have progressed from single-component, single-function uses—such as analgesia or defect filling—to multicomponent, multifunctional systems. When used as drug carriers, hydrogels have shown benefits in promoting cartilage regeneration, suppressing inflammation, and modulating immune responses (Niemeyer et al., 2022; Qiu et al., 2024; Guo et al., 2025). Contemporary smart hydrogels go further by responding to multiple endogenous cues (enzymes, reactive oxygen species, pH, temperature) and exogenous cues (light, electricity, magnetism, ultrasound, mechanical force) with spatiotemporal control. By inhibiting inflammatory pathways, maintaining mitochondrial homeostasis, repairing cartilage architecture, and restraining aberrant neurovascular ingrowth, these materials can intervene across several OA mechanisms to relieve symptoms and slow disease progression (Enayati et al., 2024; Salahuddin et al., 2024; Wu S. et al., 2024). To help readers understand the design rationale and implementation pathways of responsive hydrogels, this paper summarizes commonly used sensitive bonds and responsive units in OA, the material changes upon triggering, and the corresponding design principles (Table 1).

**TABLE 1:** Sensitive bonds and responsive units used in smart responsive hydrogels for OA applications.

Stimulus type	Sensitive bonds and responsive units	Material changes after triggering	Design principles
pH	Schiff base bonds, hydrazone bonds, acetal bonds, ketal bonds, orthoester bonds, boronate ester bonds, acid-labile ester linkages; ZIF-8, protonatable amine-containing chains, and other acid-sensitive carriers	Crosslink cleavage or network relaxation, swelling and charge switching, carrier disassembly with payload release, altered adhesion and lubrication interfaces	Align the triggering window with the magnitude of lesion acidification, and use crosslink strength and diffusion pathways to control the release tempo
ROS	thioether bonds, thioketal bonds, selenoether bonds, diselenide bonds, oxidation-cleavable boronate ester bonds, peroxalate ester bonds, disulfide bonds; oxidizable segments (PPS), antioxidant nanozymes (MnO_2_, CeO_2_, PB)	Bond cleavage or hydrophilicity–hydrophobicity switching, network degradation or swelling changes, nanozyme-assisted ROS scavenging with reshaping of the local redox state	Reduce excessive ROS while preserving physiological signaling, and maintain redox homeostasis by tuning reaction thresholds and release kinetics
Enzyme	MMP-sensitive peptide bonds, ADAMTS-sensitive peptide bonds, hyaluronidase-sensitive glycosidic bonds, cathepsin-related sensitive peptide bonds; gelatin- and collagen-derived structures, partially enzyme-hydrolyzable ester bonds	Lesion-selective degradation, pore exposure with increased permeability, payload release governed by local enzyme activity	Match enzyme profiles and align kinetics so that release follows local enzyme activity predictably while minimizing off-target burst release
Temperature	PNIPAm-based LCST phase-transition segments; Pluronic F127 thermogels; the chitosan–β-GP system; gelatin thermoreversible physical networks	Sol-to-gel transition, prolonged local retention, diffusion-restricted sustained release, and, when needed, formation of self-healing physical crosslinks	Tune the gelation temperature window to the attainable temperature rise in the joint while balancing mechanical stability and injectability
Light	photo-crosslinkable moieties (methacryloyl and other polymerizable units); photolabile groups (o-nitrobenzyl); photoisomerizable units (azobenzene); photothermal conversion fillers (MXene, MoS_2_, polydopamine, etc.)	Rapid *in situ* curing, light-triggered drug release, photothermal heating that drives phase transition or accelerates diffusion, enabling on-demand repeated activation	Match light dose to tissue-penetration depth to achieve stable and repeatable activation within the safety window
Electro	conductive networks (polypyrrole, polyaniline, PEDOT PSS); carbon-based fillers and ion-conducting components; piezoelectric units (BaTiO_3_, PVDF, short electrospun piezoelectric fibers, etc.)	Ion migration and activation of conductive pathways, piezoelectric microelectric signals generated under mechanical loading or ultrasound, potentially accompanied by accelerated local release and cellular electrostimulation effects	Convert accessible external fields into calibrated electrical signals, keep intensity and duration within tissue tolerance, and ensure fatigue stability and long-term compatibility under cyclic loading
Magnetic	magnetic nanoparticles (Fe_3_O_4_, ferrites, etc.) as magnetic-responsive units; magnetothermal materials	Magnetic guidance for localization and enrichment, magnetothermal triggering of diffusion or phase transition, magnetic-field-induced structural orientation and fine tuning	Prioritize targeting before triggering, and avoid leakage and long-term retention of magnetic materials
Ultrasound	cavitation-sensitive microbubble systems; phase-change droplet systems such as fluorocarbon phase-change cores; ultrasound-triggered microcapsule or liposome rupture	Cavitation or phase change induces structural perturbation and promotes drug release, increases local permeability, and can enable on-demand release at deep sites when needed	Calibrate ultrasound parameters with material transduction efficiency to ensure deep accessibility and uniformity
Mechanical	cyclodextrin systems; metal coordination such as catechol and iron; reversible crosslinks such as Schiff base bonds and boronate ester bonds	Shear-thinning to facilitate injection, load-triggered predictable release, self-repair to maintain network integrity, and promotion of lubrication-layer rebuilding with improved wear resistance	Co-calibrate the joint mechanical window and mechanosensitive thresholds to ensure triggerability and repeatability while balancing lubrication, wear reduction, and load-bearing stability

This review used a combined strategy of structured literature retrieval and representative study selection, with primary emphasis on publications from the past 5–10 years, while including seminal earlier studies when necessary to establish key mechanistic concepts. Guided by a mechanistic framework linking the osteoarthritis pathological microenvironment to signaling pathways, hydrogel responsiveness, and therapeutic outcomes, we conducted database searches in PubMed, Web of Science, and Scopus. Search terms encompassed osteoarthritis; smart and stimuli-responsive hydrogels; pathological microenvironment; inflammation and macrophage polarization; mitochondrial dysfunction and reactive oxygen species; autophagy and mitophagy; cartilage degeneration and regeneration; neurovascular remodeling; and delivery strategies involving exosomes and nanomaterials. We prioritized peer reviewed studies that provided mechanistic evidence and clear relevance to osteoarthritis cartilage repair, and excluded non peer reviewed reports or studies lacking mechanistic characterization.

This review surveys recent applications of stimuli-responsive hydrogels for osteoarthritis therapy. We highlight their multifaceted potential to deliver targeted anti-inflammatory effects, regulate mitochondrial function, promote cartilage repair, and modulate aberrant neurovascular invasion. We first delineate these four pathological pathways and their interactions, and we explain how hydrogel platforms achieve precise intervention by sensing and responding to endogenous and exogenous stimuli within the OA microenvironment. We then examine current challenges and outline future directions to guide rational materials design and advance clinical translation.

## 2 Suppression of inflammation mediated by smart responsive hydrogels

Chronic inflammation is a central driver across all stages of OA, delaying intrinsic joint repair and aggravating tissue injury. Targeted control of inflammation is therefore a promising therapeutic strategy. Early studies largely focused on chondrocytes and tended to overlook contributions from tissues beyond cartilage and from systemic inflammation (Berenbaum, 2013). More recent evidence shows that OA involves multiple tissues and cell subpopulations, including the synovium, subchondral bone, and immune cells, which produce inflammatory mediators that accelerate cartilage degradation. These changes are closely linked to abnormal expression of degradative enzymes, disturbance of cellular redox balance, and the emergence of a locally acidic microenvironment (Zhang et al., 2022).

In early osteoarthritis, microinjury and mechanical loading activate synoviocytes, chondrocytes, and immune cells, leading to the release of proinflammatory mediators. These mediators initiate inflammatory cascades in part by altering epigenetic regulators such as DNA methyltransferases, histone modifiers, and noncoding RNAs (Sanchez-Lopez et al., 2022; Tong et al., 2022). Cytokines then engage their cognate receptors to trigger downstream signaling. For example, interleukin-1β drives cartilage degradation via MAPK signaling, activates NF-κB, suppresses type II collagen (Col II) expression, and increases the synthesis and secretion of interleukin-6 and tumor necrosis factor-α (Wang et al., 2020; De Roover et al., 2023). As disease progresses, dysregulated epigenetic control intersects with abnormal activation of pathways that maintain cartilage homeostasis, including Wnt/β-catenin and transforming growth factor-β signaling, resulting in metabolic imbalance in chondrocytes. These cellular and molecular changes are typically accompanied by upregulation of inflammatory mediators that induce matrix metalloproteinases and ADAMTS, thereby degrading Col II and proteoglycans within the cartilage extracellular matrix and compromising articular cartilage integrity. Proinflammatory signaling also represses factors critical for matrix synthesis, including SOX9 and aggrecan, further diminishing the capacity for cartilage repair (Lu et al., 2022; De Roover et al., 2023; Du et al., 2023; Yao et al., 2023).

Persistent inflammation activates synovial fibroblasts and macrophages, resulting in synovitis and excessive generation of reactive oxygen species (ROS). Under the combined influence of inflammatory mediators and oxidative stress, chondrocytes undergo chondroaging, characterized by mitochondrial dysfunction, disordered cellular metabolism, reduced SIRT1 activity, and impaired autophagy. These changes diminish cellular viability and, through the senescence-associated secretory phenotype (SASP), activate neighboring cells and maintain a chronically inflamed joint microenvironment (Rezuş et al., 2019; Chen et al., 2020; Xu X. et al., 2025). In parallel, dense infiltration of T lymphocytes and macrophages within the synovium elevates vascular endothelial growth factor (VEGF) and nerve growth factor (NGF) expression, promoting aberrant angiogenesis and sprouting of sensory nerve fibers that intensify pain (Hu Y. et al., 2021; Zhao L. et al., 2024).

To achieve efficient and precise local control of inflammation, current research emphasizes advanced intraarticular delivery platforms. Hydrogels are a promising option because of their biocompatibility, tunable mechanics, and injectability (Qian et al., 2025). Smart hydrogels can provide on demand release triggered by features of the OA microenvironment or by exogenous cues, thereby improving localization and durability of anti-inflammatory agents. In doing so, they help attenuate chronic inflammation within the joint and may interrupt the cycle of inflammation-driven tissue damage.

### 2.1 Endogenous stimuli-responsive hydrogels targeting inflammatory responses in osteoarthritis

Alterations in biological and physiological parameters guide both diagnosis and the development of hydrogels that respond to endogenous stimuli. In osteoarthritic joints, features of the local microenvironment, such as acidic synovial fluid, accumulation of reactive oxygen species, and elevated expression of matrix metalloproteinases and proinflammatory cytokines, accelerate cartilage degeneration and synovitis (Yao et al., 2023). Leveraging these signatures, investigators have engineered endogenous stimuli-responsive hydrogels that sense disease cues to enable localized, controlled release and precise modulation of inflammatory pathways (Table 2).

**TABLE 2:** Endogenous stimuli-responsive hydrogels targeting inflammation.

Endogenous stimuli	Material components	Responsive mechanism	Bioactive agent	Effects	References
pH	GelMA, Itaconate (IA), ZIF-8 Metal-Organic Framework (ZIF-8 MOFs)	ZIF-8 MOFs	IA	attenuate oxidative stress and downregulate TNF-α and IL-6 expression	Yu et al. (2023)
	oxidized hyaluronic acid (OHA), quaternized chitosan (QCS), diacerein (DIA), nano hydroxyapatite (NHAp)	Schiff base bonds between OHA and OCS	DIA	suppress inflammatory cytokines, downregulate iNOS expression, and decrease NO production	Zhang et al. (2025a)
	methylcellulose (MC), hyaluronic acid (HA), carboxymethyl chitosan (CCM), quercetin (QUE), ZIF-8 MOFs	ZIF-8 MOFs	QUE, Zn^2+^	scavenge ROS, promote macrophage polarization toward M2, upregulate CD206 and downregulate CD86, and reduce the expression of inflammatory markers	Zhang et al. (2025b)
	tea polyphenol nanoparticles (TPNPs), carboxy PTIO	acid-triggered protonation of the carboxylate in carboxy PTIO.	TPNPs, PTIO	synergistically scavenge ROS and NO and, by suppressing ROS/NF-κB and iNOS/NO/Caspase 3 signaling, effectively reduce inflammatory cytokine release	Ding et al. (2025)
ROS	PVA, HA-PBA, PEI-PEG, Fe_3_O_4_NPs, MMP-13 siRNA	boronate ester linkage between PBA and diols	MMP-13 siRNA	suppress MMP-13 and IL-1β expression in chondrocytes under inflammatory conditions and effectively alleviate synovitis	Wang et al. (2025c)
	synovium-derived exosomes enriched in SOD3 (S-EXOs), GelMA, PDA	PDA coating	S-EXOs	reduce intracellular ROS and mitoROS and effectively suppress MMP-13 and ADAMTS5 expression	Cao et al. (2024)
	Prussian blue (PB), Alg, HA	PB metal organic framework	PB	scavenge ROS, release oxygen, and downregulate the expression of MMP-13, IL-1β, and TNF-α	Peng et al. (2025)
Enzyme	Chs, SerMA, DPG peptide sequence, 78c, folic acid (FA), liposomes	enzymatic cleavage of the DPG peptide by MMP-9	78c, FA	inhibit CD38 and downregulate PI3K/AKT signaling and inflammation-related gene expression, thereby ameliorating chondrocyte dysfunction	He et al. (2025)
	circ-srIκBα, lipid nanoparticles (LNPs), SF, HA, CS, substrate peptide cleavable by MMP-13	substrate peptide cleavable by MMP-13	srIκBα	inhibit NF-κB pathway activity, lower local joint inflammation, and reverse the gene expression profile associated with OA; this effect was validated in human OA cartilage tissue	Li et al. (2025b)
	SAC4A-MA, HA-MA, substrate peptide cleavable by MMP-13, hydroxychloroquine (HCQ)	substrate peptide cleavable by MMP-13	HCQ	suppress macrophage inflammatory responses, mitigate oxidative stress, and downregulate HIF-1α and inflammatory cytokine expression	Zhou et al. (2024b)
Temperature	AMP, MCPs, chitosan, β-GP, radioactive lutetium-177 (^177^Lu)	chitosan, β-GP	^177^Lu	suppress synovitis progression, promote macrophage polarization, reduce proinflammatory cytokine levels, decrease iNOS and increase CD206 expression	Liu et al. (2025b)
	ADSC-derived exosomes (EVs), celecoxib, Pluronic F127, HA	Pluronic F127	EVs, celecoxib	promote macrophage polarization, lower proinflammatory cytokines, alleviate synovitis, and improve the inflammatory microenvironment of the knee joint	Fu et al. (2025)
	Ozone (O_3_), tributylamine fluoride, fluorinated hyaluronic acid (FHA), D-mannose, hydroxypropyl chitosan (HPCH)	HPCH	O_3_, D-mannose	reduced VEGF and overall inflammatory levels, alleviated synovitis, and attenuated cartilage destruction	Wu et al. (2024a)

#### 2.1.1 pH responsive hydrogels

In healthy joints, the microenvironment is mildly alkaline (pH 7.4–7.8), which supports chondrocyte metabolism and joint lubrication. Inflammatory mediators increase cellular activity and oxygen consumption while shifting metabolism toward anaerobic glycolysis, thereby elevating lactate production. At the same time, reduced activity of proton coupled monocarboxylate transporters (MCTs) limits lactate efflux, and the avascular nature of cartilage favors accumulation of acidic metabolites; as a result, synovial pH can fall to 6.2–6.6 (Zheng et al., 2021; Tan et al., 2022; Arra and Abu-Amer, 2023). pH responsive hydrogels detect changes in hydrogen ion concentration through reversible protonation and deprotonation. The resulting shifts in ionization alter network charge density and osmotic pressure, producing reversible swelling or deswelling. For example, contraction at lower pH when charges are neutralized and expansion at higher pH due to electrostatic repulsion (Lim et al., 2014; Salahuddin et al., 2024). Leveraging the acidic milieu in OA, researchers introduce acid sensitive groups (amino, carboxyl, thiol) or protonatable and acid labile linkages (esters, Schiff bases, vinyl ethers) into drug loaded matrices to achieve condition triggered degradation and precise, on demand release of anti-inflammatory agents (Ding et al., 2022).

**FIGURE 1:**
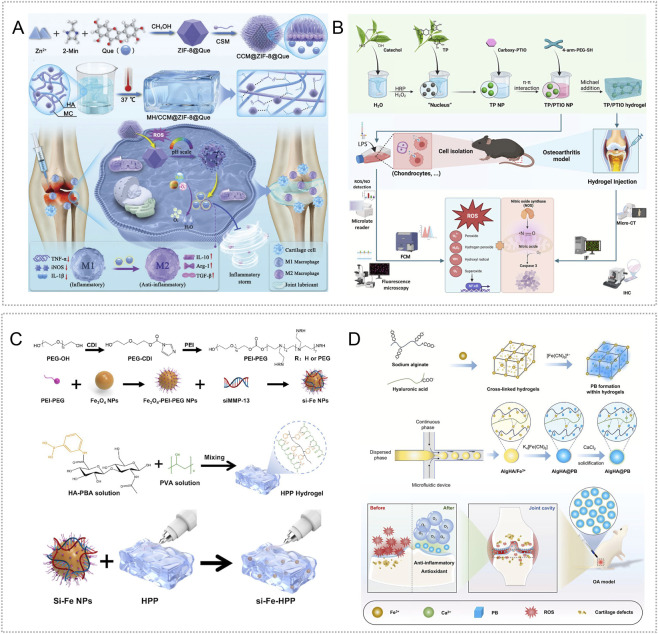
pH and ROS responsive hydrogels targeting inflammatory responses. **(A)** A dual pH/ROS responsive platform formed by loading Que onto ZIF-8 and embedding it in an MH/CCM hydrogel enables on demand release of Que and Zn^2+^, scavenges ROS, induces macrophage M2 polarization, and suppresses inflammation. Reproduced with permission from Ref. (Zhang H. et al., 2025), Copyright 2025, Wiley-VCH GmbH. **(B)** A pH/ROS dual responsive hydrogel prepared by thiol–thiol crosslinking of tea polyphenol nanoparticles and carboxy-PTIO using 4-arm-PEG-SH scavenges ROS and NO and inhibits inflammatory signaling, thereby regulating oxidative stress and inflammation. Reproduced with permission from Ref. (Ding et al., 2025), Copyright 2025, Elsevier. **(C)** MMP-13 siRNA carried by PEI–PEG–modified Fe_3_O_4_ nanoparticles and embedded in a hydrogel crosslinked through boronate bonds between PBA-modified hyaluronic acid and PVA yields a ROS responsive system that targets and suppresses inflammation. Reproduced with permission from Ref. (Wang Q. et al., 2025), Copyright 2025, Springer Nature. **(D)** Schematic of AlgHA@PB hydrogel fabrication and its mechanism of anti-inflammatory and antioxidant action in OA treatment. Reproduced with permission from Ref. (Peng et al., 2025), Copyright 2025, Elsevier.

Leveraging the large surface area, tunable functionality, high drug-loading capacity, and pH sensitivity of ZIF-8 metal–organic frameworks (MOFs), Yu et al. fabricated GelMA-based hydrogel microspheres (IA-ZIF-8@HMs) by encapsulating IA-ZIF-8 nanoparticles. The microspheres enabled acid-triggered release of IA, attenuated oxidative stress, and lowered TNF-α and IL-6 levels, demonstrating strong anti-inflammatory and antioxidant activity (Yu et al., 2023). Li et al. constructed an injectable pH responsive hydrogel (DIA-MS@gel) via Schiff base crosslinking of oxidized hyaluronic acid (OHA) and quaternized chitosan (QCS) that encapsulated DIA sustained release microspheres (DIA-MS). *In vitro*, this system suppressed inflammatory cytokines as well as iNOS and NO expression (Zhang B. et al., 2025). Zhang et al. further developed a pH/ROS dual responsive composite hydrogel (MH/CCM@ZIF-8@Que) by loading quercetin (Que) onto ZIF-8 and embedding it in a thermosensitive carboxymethyl chitosan–gelatin matrix, thereby combining lubrication with smart responsiveness. In acidic, ROS-rich joint conditions, the material released quercetin and Zn^2+^ in a controlled manner, scavenged ROS, promoted macrophage M2 polarization with CD206 upregulation and CD86 downregulation, enhanced bone-marrow mesenchymal stem-cell proliferation and osteogenic potential, and reduced inflammatory markers. In a monoiodoacetic-acid (MIA) induced rat model, it significantly lowered friction coefficients and alleviated synovitis (Zhang H. et al., 2025) (Figure 1A).

In addition, Ding et al. designed a hydrogel platform by linking tea-polyphenol antioxidant nanoparticles (TPNPs) with the nitric-oxide scavenger carboxy-PTIO through thiol–thiol crosslinking of 4-arm-PEG-SH. The system provided dual responsive release under low pH and high ROS. At pH 6.0, protonation of carboxy-PTIO weakened π–π interactions with TPNPs, enabling controlled release of tea polyphenols and carboxy-PTIO. In a papain-induced mouse OA model, TP/PTIO nanoparticles synergistically eliminated ROS and NO and reduced inflammatory cytokines by inhibiting ROS/NF-κB and iNOS/NO/Caspase-3 signaling (Ding et al., 2025) (Figure 1B).

In summary, pH responsive hydrogels leverage the mildly acidic milieu of osteoarthritic joints to achieve lesion preferential drug release via pH driven swelling or deswelling and cleavage of acid labile linkages, thereby supporting anti-inflammatory and antioxidant therapy. Design should match ionizable motifs or acid sensitive chemistries to the OA pH range and tune crosslink density to remain stable at physiological pH yet respond under mild acidity. Pairing pH triggering with functions such as ROS scavenging and inflammatory pathway suppression may further enhance local efficacy and durability. Key challenges remain long term intra articular stability, sufficient tissue penetration, and sustained modulation of the pathological microenvironment.

#### 2.1.2 ROS responsive hydrogels

Reactive oxygen species (ROS) act as key secondary messengers that activate multiple transcription factors and amplify inflammatory responses (Manoharan et al., 2024). In osteoarthritis (OA), ROS function both as products of inflammation and as upstream drivers, creating a feedback loop between oxidative stress and inflammation. Excess ROS damage mitochondria, DNA, and proteins, leading to chondrocyte injury and, ultimately, matrix degradation and joint degeneration (Blanco et al., 2018; Wu et al., 2023; Li et al., 2025a). It is important to note that ROS are not solely harmful byproducts. At physiological levels, they serve as signaling mediators that regulate cell proliferation and differentiation, stress adaptation, and tissue repair. Accordingly, therapy should aim to restore redox homeostasis by curbing excessive ROS at pathological sites while preserving basal ROS signaling needed to support repair (Sies and Jones, 2020). ROS-responsive hydrogels exploit the high chemical reactivity of ROS through selective reactions with built-in functional groups. Common ROS-sensitive motifs include sulfides, thioacetals, selenides or selenoacetals, and boronate esters; oxidation of these units triggers predictable changes in network structure and function (Liu J. et al., 2023).

Wang et al. loaded MMP-13 siRNA onto Fe_3_O_4_ nanoparticles coated with a PEI–PEG shell to create a nucleic-acid carrier (si-Fe NPs) that protected siRNA from degradation and improved gene silencing. Encapsulation within a hyaluronic acid hydrogel modified with phenylboronic acid and crosslinked with poly (vinyl alcohol) through boronate ester bonds yielded a dual-function material (si-Fe-HPP) with ROS sensitivity and RNA interference activity. In a mouse model of OA induced by destabilization of the medial meniscus, hydrogen peroxide triggered siRNA release, which reduced MMP-13 and IL-1β expression in inflamed chondrocytes and mitigated synovitis, osteophyte formation, and subchondral sclerosis (Wang Q. et al., 2025) (Figure 1C).

Exosomes are central mediators of intercellular communication and can reprogram inflamed microenvironments by ferrying anti-inflammatory cargo (Peng et al., 2023). Using single-cell RNA sequencing, Cao et al. identified superoxide dismutase 3 (SOD3) as a candidate node in cartilage–synovium crosstalk and created an injectable microsphere system (GM@PDA@S-EXO) by stably loading SOD3-rich synovial exosomes into gelatin–polydopamine hydrogel microspheres. In ROS-rich conditions, the microspheres released exosomes, lowered intracellular ROS and mitochondrial ROS, and suppressed MMP-13 and ADAMTS5 expression *in vitro* (Cao et al., 2024). In addition, Prussian blue (PB) nanozymes display multiple enzyme-like activities resembling superoxide dismutase, catalase, and peroxidase (Cho et al., 2023). Peng et al. embedded PB nanozymes in alginate–hyaluronic acid hydrogel microspheres (AlgHA@PB) using microfluidics. In OA-mimicking inflammatory conditions, the platform scavenged ROS and released oxygen, downregulated MMP-13, IL-1β, and TNF-α, and, in monoiodoacetate-induced rat OA, delayed cartilage degeneration while decreasing pro-inflammatory cytokines and increasing anti-inflammatory mediators (Peng et al., 2025) (Figure 1D).

It is worth emphasizing that although ROS-responsive hydrogels can mitigate OA inflammation, their long-term metabolic safety and the risk of disrupting physiological ROS signaling warrant systematic evaluation. These systems should be designed to restore redox homeostasis by tuning activation thresholds to the local ROS range at the lesion site and aligning release kinetics accordingly. Such calibration would preferentially remove excess ROS while preserving repair-relevant signaling, enabling more stable, controllable, and physiologically meaningful therapeutic effects.

#### 2.1.3 Enzyme responsive hydrogels

In the osteoarthritic inflammatory microenvironment, mediators activate NF-κB and MAPK signaling and sustain high expression of matrix-degrading enzymes, notably MMPs (including MMP-13) and ADAMTS. This enzymatic activity is a major driver of extracellular-matrix (ECM) breakdown and structural failure of the joint (Yao et al., 2023; Sengprasert et al., 2024). Enzyme responsive hydrogels address this feature by embedding sequences or crosslinks—such as peptide bonds, esters, and gelatin-derived motifs—within the network that are selectively cleaved by MMPs or ADAMTS, thereby enabling site specific degradation and on-demand drug release at lesions with elevated enzyme levels (Xiao and Huang, 2024).

**FIGURE 2:**
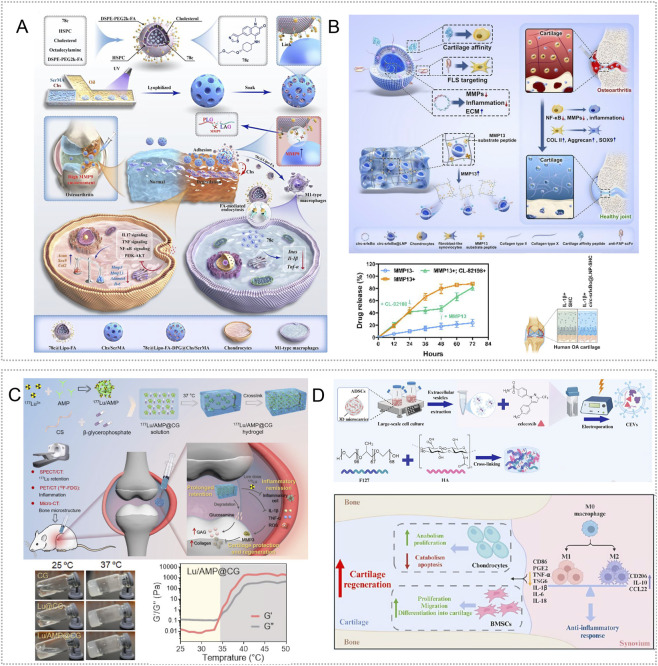
Enzyme responsive and thermosensitive hydrogels targeting inflammatory responses. **(A)** LFDCS injectable microspheres with a chondroitin sulfate/methacrylated sericin core and a surface bearing 78c-loaded folate liposomes that display the DPG peptide. MMP-9 recognition and cleavage trigger on demand release; folate targets M1 macrophages and 78c suppresses inflammation by downregulating PI3K/AKT. Reproduced with permission from Ref. (He et al., 2025), Copyright 2025, Springer Nature. **(B)** A composite hydrogel formed by crosslinking sericin, hyaluronic acid, and chondroitin sulfate with an MMP-13–cleavable peptide, loaded with circ-srIκBα encapsulated in modified lipid nanoparticles. It selectively targets chondrocytes and fibroblast-like synovial cells to inhibit NF-κB signaling; efficacy was validated in human OA cartilage tissue. Reproduced with permission from Ref. (Li et al., 2025b), Copyright 2025, Elsevier. **(C)** ^177^Lu/AMP@CG, a thermosensitive chitosan/β-glycerophosphate gel that rapidly solidifies at body temperature, delivers continuous local β radiation, scavenges ROS, promotes macrophage polarization toward M2, lowers inflammatory mediators, and protects cartilage. Reproduced with permission from Ref. (Liu P. et al., 2025), Copyright 2025, Wiley-VCH GmbH. **(D)** CEVs@F127–HA thermosensitive hydrogel uses an F127–HA matrix with celecoxib encapsulated in engineered exosomes; the exosome–drug combination promotes M2 polarization, decreases inflammatory cytokines, and remodels the joint regenerative microenvironment. Reproduced with permission from Ref. (Fu et al., 2025), Copyright 2025, American Chemical Society.

He et al. engineered injectable hydrogel microspheres (LFDCS) based on chondroitin sulfate and methacrylated sericin. Surface functionalization employed DPG-modified liposomes (DSPE-PEG2k-GPLGLAGQC) co-loaded with 78c and folic acid (78c@Lipo-FA) for targeted delivery. MMP-9 cleavage of the DPG sequence triggered 78c release; folate then directed uptake via folate receptors on M1 macrophages, where 78c suppressed proinflammatory activity and limited cartilage catabolism. Concurrently, released chondroitin sulfate supported chondrocyte function. RNA-seq indicated that LFDCS mitigated chondrocyte dysfunction by inhibiting CD38 and downregulating PI3K/AKT signaling and inflammation-related genes. In an ACLT rat model, LFDCS reduced M1-driven synovial inflammation (He et al., 2025) (Figure 2A). In addition, Li et al. developed a circ-srIκBα@LNP-SHC platform in which the super-repressor circRNA was packaged in modified lipid nanoparticles and embedded in a composite hydrogel of sericin, hyaluronic acid, and chondroitin sulfate crosslinked with an MMP-13 sensitive peptide. The system targeted chondrocytes and fibroblast-like synoviocytes, inhibited NF-κB activity, and lowered local inflammatory burden. In papain-induced OA, it decreased inflammatory cytokines and matrix-degrading enzymes and reversed OA-associated gene expression; similar benefits were observed *ex vivo* in human OA cartilage (Li et al., 2025b) (Figure 2B).

Zhou et al. constructed an enzyme responsive delivery system by integrating MMP-13 sensitive peptides bearing terminal thiols into a hydrogel network (HAM-SA@HC) and loading hydroxychloroquine. Enzymatic cleavage enabled controlled release, while the matrix scavenged reactive oxygen species and dampened macrophage activation, reducing HIF-1α and proinflammatory cytokines (Zhou T. et al., 2024). In a complementary “logic-gate” design, Li et al. created KM13E@PGE microspheres in which an MMP-13 responsive peptide hydrogel rapidly released IL-10^+^EVs to initiate anti-inflammation, followed by slower release of SOX9^+^EVs from PEGDA/GelMA microspheres to promote ECM repair, achieving a sequential “anti-inflammation first, repair second” regimen (Li et al., 2024).

Enzyme-responsive hydrogels enable lesion-selective release in osteoarthritis to curb inflammation, limit matrix degradation, and support cartilage repair. Performance, however, is shaped by spatiotemporal variability in enzyme expression, interpatient differences, and stage-dependent shifts during disease progression. Design should therefore tune activation thresholds to lesion-site enzyme activity and match release kinetics accordingly. Cleavable motifs can be tailored to disease-relevant enzymes such as MMPs and ADAMTS, while crosslink density, substrate accessibility, and diffusion pathways are jointly optimized. Together, these parameters link local enzyme activity to release rate, reducing off-site leakage and minimizing burst release into the joint cavity.

#### 2.1.4 Temperature responsive hydrogels

Temperature responsive hydrogels achieve precise temperature sensitivity through polymer critical-solution behavior, dynamic regulation of crosslink density, and shifts in hydrophobic–hydrophilic balance. They undergo sol–gel transition at body temperature without external triggers (Sun Z. et al., 2020). Natural matrices include chitosan, gelatin, cellulose, and hyaluronic acid, whereas synthetic options include poly(N-isopropylacrylamide), poloxamers, and polyethylene-glycol–based systems (Chen H. et al., 2024).

Radioactive synovial repair (RSO) delivers radioisotopes intraarticularly so that emitted β radiation suppresses synovial hyperplasia and limits collateral cartilage and bone damage (Kampen et al., 2022). Liu et al. encapsulated lutetium-177 in adenosine-monophosphate–based metal–organic coordination polymers (^177^Lu/AMP MCP) and embedded these within a chitosan/β-glycerophosphate thermoresponsive hydrogel (^177^Lu/AMP@CG). The formulation remained fluid at room temperature and gelled rapidly after injection at body temperature. Local release of ^177^Lu provided synovial radiotherapy, while the CG matrix attenuated synovitis by promoting macrophage polarization from M1 to M2, lowering proinflammatory cytokines, decreasing iNOS, increasing CD206, and scavenging reactive oxygen species. In an ACLT induced rat model, systemic distribution was minimal and no significant *in vivo* toxicity was observed, indicating favorable radiologic safety (Liu P. et al., 2025) (Figure 2C). Fu et al. increased exosome yield and potency by culturing adipose-derived mesenchymal stem cells on three-dimensional microcarriers, then loaded celecoxib into the vesicles by electroporation to generate engineered exosomes (CEVs) with dual anti-inflammatory and chondrogenic activity. For delivery, they formulated a thermoresponsive carrier with F127 and hyaluronic acid (CEVs@F127-HA). *In vitro* and in MIA induced murine OA, the system promoted macrophage polarization from M1 to M2, reduced proinflammatory cytokines, shifted the joint milieu toward an anti-inflammatory state, preserved chondrocyte metabolic homeostasis, and supported BMSC chondrogenic differentiation (Fu et al., 2025) (Figure 2D).

Ozone (O_3_) has antioxidant, anti-inflammatory, and immunomodulatory effects (Migliorini et al., 2020). Wu et al. encapsulated O_3_ in nanoparticles composed of tributylfluoride and fluorinated hyaluronic acid, then conjugated D-mannose to hydroxypropyl chitosan via Schiff-base chemistry to obtain MHPCH. Embedding O_3_ nanoparticles in MHPCH produced a thermoresponsive composite hydrogel (O_3_NPs@MHPCH) that lowered VEGF and inflammatory cytokines, alleviated synovitis, reduced cartilage injury, and inhibited subchondral trabecular remodeling (Wu H. et al., 2024). Temperature responsive hydrogels combine injectability with rapid *in situ* gelation to enhance intra-articular retention and provide localized anti-inflammatory, immunomodulatory effects in osteoarthritis. Key design requirements include room-temperature flowability, fast gelation at physiological temperature, and network integrity under synovial dilution and mechanical loading. Gelation should be tuned to the joint’s operative temperature range, with crosslink density and hydrophobic interactions co-optimized to limit temperature-driven release drift and prevent premature destabilization.

### 2.2 Exogenous stimuli-responsive hydrogels targeting inflammatory responses in osteoarthritis

Exogenous Stimuli-responsive hydrogels can trigger drug release or alter material properties at defined times and sites by incorporating external physical inputs such as light, magnetic fields, ultrasound, or mechanical force, thereby modulating local inflammation with high precision. In the osteoarthritic microenvironment, these externally activated systems regulate key nodes, including cytokine expression, reactive oxygen species (ROS) levels, and stress induced apoptosis while offering on demand activation and spatial targeting (Table 3).

**TABLE 3:** Exogenous stimuli-responsive hydrogels targeting inflammation.

Exogenous stimuli	Material components	Responsive mechanism	Bioactive agent	Effects	References
Light	GelMA, WYRGRL, exosomes, LRRK2-IN-1	UV crosslinking	WYRGRL, LRRK2-IN-1	suppress expression of OA-associated inflammatory and immune genes, reverse transcriptomic changes induced by IL-1β, and exhibit chondrocyte targeting capability	Wan et al. (2023)
	MoS_2_, capsaicin, curcumin, platelet-derived exosomes (pEVs), F127, MC	photothermal effect of MoS_2_	MoS_2_, capsaicin, curcumin, pEVs	suppress M1 polarization, promote M2 polarization, scavenge ROS, and reduce inflammation; inhibit angiogenesis and ameliorate the hypoxic microenvironment	Chen et al. (2025a)
	Mo_2_Ti_2_C_3_ MXene, gelatin, chitosan	photothermal effect of Mo_2_Ti_2_C_3_ Mxene	Mo_2_Ti_2_C_3_ Mxene	downregulate M1 macrophage–associated cytokines IL-1β and IL-6, upregulate M2-associated cytokines TGF-β and IL-10, and lower ROS levels *in vivo*	Wang et al. (2025b)
Magnetic	HAMA, CSMA, Fe_3_O_4_@MgSiO_3_ nanoparticles, sodium diclofenac (DS)	Fe_3_O_4_@MgSiO_3_ magnetic nanoparticles	DS, Fe_3_O_4_@MgSiO_3_	inhibit inflammatory signaling, promote macrophage polarization toward M2, and lower proinflammatory cytokine levels	Yang et al. (2024b)
Ultrasound	hydrocortisone, F127, HA, gelatin, surfactants (Tween®60, Capryol®, Span®20 and Brij®35)	disrupt physical interactions through cavitation and temperature elevation	hydrocortisone	reduce inflammatory cytokine levels and suppress downstream inflammatory and catabolic pathways	Jahanbekam et al. (2023)
Mechanical	a conjugate of hyaluronic acid sulfate and sodium diclofenac (SYN321), OA-on-chip platform(uBeat® MultiCompress)	hydrolysis of ester bonds on modified HA	sodium diclofenac	downregulate inflammatory markers and reduce the activity of matrix-degrading enzymes, thereby inhibiting ECM degradation	Palma et al. (2024)
	Mg^2+^, Dimethyloxalylglycine (DMOG), HAMA, PBA, High-glucose DMEM, BMSCs	PBA can form reversible boronate ester bonds with polyhydroxy groups	PBA and polyhydroxy groups	This approach ensures cell retention and functional activity at the injury site. The system markedly suppresses joint inflammation, enhances cartilage regeneration, and improves joint function	Gao et al. (2025)

**FIGURE 3:**
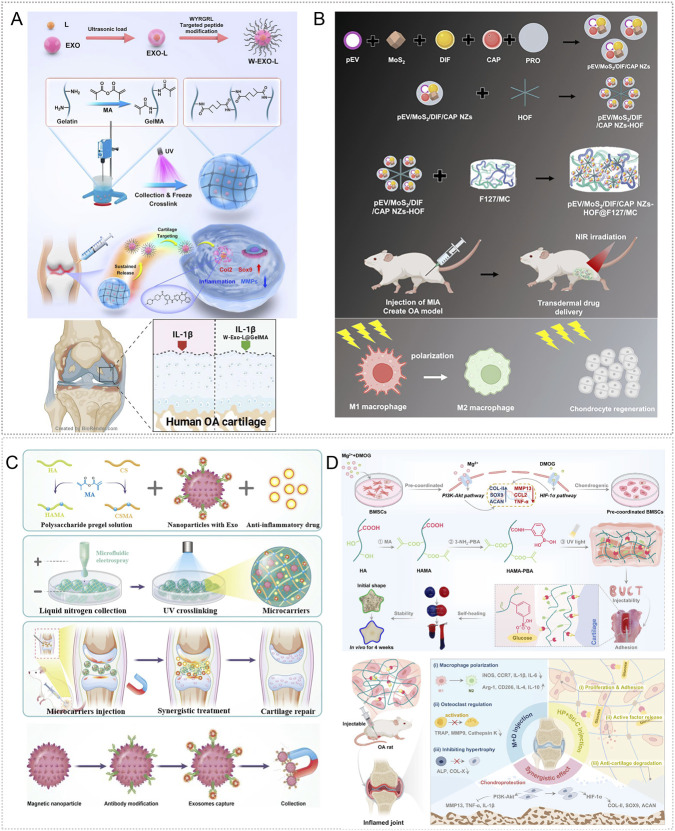
Exogenous stimuli-responsive hydrogels targeting inflammatory responses. **(A)** UV crosslinked W-Exo-L@GelMA encapsulating engineered exosomes (W-Exo) and LRRK2-IN-1; the system gels under light and synergistically reverses IL-1β–induced pathology through anti-inflammatory and immunomodulatory actions, with efficacy validated in human cartilage tissue. Reproduced with permission from Ref. (Wan et al., 2023), Copyright 2023, Springer Nature. **(B)** pEV/MoS_2_/DIF/CAP NZ-HOF@F127/MC platform with NIR triggered release that reduces inflammation, modulates immune responses, alleviates hypoxia, and inhibits angiogenesis, thereby remodeling the joint microenvironment. Reproduced with permission from Ref. (Chen C.-K. et al., 2025), Copyright 2025, Elsevier. **(C)** Porous magnetic polysaccharide microcarriers based on HAMA and CSMA, embedded with Fe_3_O_4_@MgSiO_3_ nanoparticles and sodium diclofenac; magnetic field guidance enables lesion targeting, while enrichment of MSC exosomes and drug synergy suppress inflammatory signaling. Reproduced with permission from Ref. (Yang L. et al., 2024), Copyright 2024, American Chemical Society. **(D)** Mg^2+^/DMOG pre-coordinated BMSCs encapsulated in HAMA-PBA dynamic borate hydrogels form a self-adaptive cell delivery system. This injectable formulation supports *in situ* cell adhesion and self-healing, while promoting macrophage anti-inflammatory polarization, suppressing osteoclast activity, and activating HIF-1α to mimic hypoxia. Collectively, it attenuates OA inflammation and cartilage degeneration and improves cartilage repair and functional recovery. Reproduced with permission from Ref. (Gao et al., 2025), Copyright 2025, Elsevier.

Light is an attractive energy source because it allows flexible control, high spatiotemporal resolution, and convenient operation (Rapp and DeForest, 2020). Common excitation bands include ultraviolet (UV, 200–400 nm), visible (VIS, 400–700 nm), and near infrared (NIR, 700–1300 nm), enabling depth dependent stimulation (Xing et al., 2022). Wan et al. engineered a UV crosslinked GelMA spherical hydrogel (W-Exo-L@GelMA) that encapsulated engineered exosomes modified with the chondro affinity peptide WYRGRL and the anti-inflammatory inhibitor LRRK2-IN-1. The platform suppressed inflammation and immune related genes associated with OA, reversed interleukin-1β induced transcriptomic changes, and showed strong chondrocyte targeting. In destabilization of the medial meniscus (DMM) mouse OA and in human OA cartilage cultures, it demonstrated robust anti-inflammatory and cartilage repairing effects (Wan et al., 2023) (Figure 3A). The NIR band combines moderate energy with deeper tissue penetration and favorable biosafety (Xing et al., 2022). Chen et al. created an injectable F127–methylcellulose hydrogel that co delivered nanozymes and anti-inflammatory agents (MoS_2_, capsaicin, curcumin) together with platelet derived exosomes, yielding the pEV/MoS_2_/DIF/CAP NZ-HOF@F127/MC system. Upon NIR illumination, the platform released cargo, inhibited M1 and promoted M2 macrophage polarization, scavenged ROS, reduced inflammation, suppressed angiogenesis, and improved hypoxia in monoiodoacetate (MIA) rat OA (Chen C.-K. et al., 2025) (Figure 3B). In related work, Wang et al. formulated a Mo_2_Ti_2_C_3_ MXene gelatin–chitosan hydrogel; 808 nm NIR irradiation converted light to heat via MXene nanosheets, elevating local temperature to activate function, reduce ROS, suppress pro inflammatory factors, and promote cartilage regeneration (Wang H. et al., 2025).

Magnetic fields provide a controllable, non-ionizing stimulus with good tissue penetration and are widely used in medicine (Ramos-Sebastian et al., 2022). Magnetoresponsive hydrogels typically embed magnetic nanoparticles (e.g., Fe_3_O_4_, Co_3_O_4_, NiO) within a polymer network; static or alternating magnetic fields induce nanoparticle responses that enable controlled drug release (Hao and Mao, 2023). Using oxidized hyaluronic acid (HAMA) and chondroitin sulfate methacrylate (CSMA), investigators produced porous magnetic polysaccharide microcarriers by microfluidic electrospray and gelation. These carriers encapsulated Fe_3_O_4_@MgSiO_3_ nanoparticles capable of binding mesenchymal stem cell exosomes (MSC-Exo) and co loading sodium diclofenac. External magnetic guidance positioned the carriers at lesions, prolonging intraarticular retention of both exosomes and drug. *In vitro* and in ACLT rat OA, combined diclofenac and MSC-Exo suppressed inflammatory signaling, induced M2 polarization, reduced cytokines, and protected cartilage (Yang L. et al., 2024) (Figure 3C).

Ultrasound influences tissues through cavitation, mechanical compression, acoustic streaming, and localized heating; orthopedic applications often use 3–20 kHz low frequency regimens (Zhou Y. et al., 2022). Ultrasound responsive hydrogels change structure during insonation to enable precise, on-site release. Jahanbekam et al. formulated an F127–hyaluronic acid–gelatin injectable hydrogel loaded with mixed micelles of hydrocortisone and surfactants. The gel solidified at body temperature and provided sustained hydrocortisone release under 160 W, 35 kHz low frequency ultrasound, thereby suppressing synovitis in OA models (Jahanbekam et al., 2023).

Mechanical factors are also relevant. Healthy cartilage exhibits extremely low shear friction, whereas elevated shear accelerates wear, induces degradative enzymes, and establishes a positive feedback that promotes OA (Szabo et al., 2023; Ristaniemi et al., 2024). Mechanically responsive hydrogels that sense shear during joint motion to trigger release and reorganize to form durable lubricating layers may slow disease progression (Ma P. et al., 2022). SYN321, a hyaluronic acid sulfate–sodium diclofenac conjugate for sustained intraarticular NSAID delivery and lubrication, was evaluated on an OA-on-chip platform (uBeat® MultiCompress) capable of up to 30% compressive strain. This three layer device, consisting of a fibrin hydrogel cell layer, a mechanical actuation layer, and a glass cover, recapitulated joint dynamics and showed that SYN321 downregulated inflammatory markers, reduced matrix metalloproteinase activity, limited extracellular matrix degradation, and preserved microtissue architecture (Palma et al., 2024). In recent years, the multifunctional therapeutic potential of Mg^2+^ in orthopedic tissue engineering has attracted substantial attention. Wang et al. reported that Mg^2+^ incorporated nanocomposites hold considerable promise for osteoarthritis management and cartilage regeneration (Wang X. et al., 2025). Additionally, Gao et al. preconditioned BMSCs with Mg^2+^ and the hypoxia mimetic DMOG to enhance their immunomodulatory and chondrogenic capacity, and then encapsulated the cells in a HAMA-PBA hydrogel. HAMA-PBA forms reversible boronate ester bonds with polyhydroxy moieties in the tissue milieu, which confers shear thinning injectability, self-healing driven by stress relaxation, and adaptive mechanical behavior that promotes adhesion to cartilage surfaces and conformability *in situ*. These properties improve local cell retention and sustain therapeutic function. Mechanistically, Mg^2+^ promotes macrophage polarization toward an anti-inflammatory phenotype and suppresses osteoclast activation, thereby preserving subchondral bone integrity through PI3K-Akt signaling. DMOG stabilizes and activates HIF-1α to emulate the hypoxic cartilage microenvironment, supporting chondrocyte repair and extracellular matrix synthesis. *In vitro*, this platform enhanced BMSC chondrogenic differentiation and upregulated key chondrogenic markers. In rat osteoarthritis models, it attenuated joint inflammation, mitigated cartilage degeneration, and improved joint function (Gao et al., 2025) (Figure 3D).

Exogenous stimuli-responsive hydrogels enable precise drug delivery and microenvironmental intervention to regulate OA inflammation. Their design depends on a quantifiable coupling between controllable energy input and key inflammatory nodes. Within clinically feasible ranges of light, magnetic, acoustic, and mechanical parameters, activation thresholds and release kinetics should be tuned to achieve predictable dose-dependent release while preserving joint retention, lubrication, and tissue safety margins. Many platforms, however, still require external devices and remain constrained by limited penetration depth. Integrating penetration and positioning into a unified design will be critical to simplify operation while improving regional selectivity and translational feasibility.

## 3 Modulation of mitochondrial function mediated by smart responsive hydrogels

Mitochondrial dysfunction is a major driver of osteoarthritis (OA) onset and progression and may precede overt cartilage loss. It contributes through several routes that are integral to joint degeneration, including hypoxia related signaling in synovial lining cells, impaired chondrocyte biosynthesis, and dysregulated growth responses (Hu et al., 2020; Liu et al., 2022). Chondrocytes are central to the balance between synthesis and degradation of the extracellular matrix (ECM). Mitochondrial injury elevates MMP-3, MMP-13, nitric oxide, and proinflammatory cytokines, which accelerates ECM breakdown and cartilage degeneration (Molnar et al., 2021). Chondrocytes from patients with OA commonly show a reduced mitochondrial membrane potential, lower ATP production, and increased reactive oxygen species, changes that intensify oxidative stress and apoptosis and further promote matrix loss (Charlier et al., 2016). Aging, infection, nutrient deficiency, and genetic variants are closely linked to mitochondrial impairment in OA; for example, abnormal expression of the autophagy receptors Parkin and p62 and dysregulation of the PGC-1α/NRF-1 axis reduce mitochondrial biogenesis and compromise quality control (Kim et al., 2021; D’Amico et al., 2022). Overall, OA associated mitochondrial dysfunction encompasses depleted ATP, oxidative stress, disturbed dynamics and metabolism, morphological abnormalities, and disrupted calcium homeostasis, which together trigger a cascade that culminates in apoptosis and accelerates cartilage degeneration.

With advances in stem cell therapy and biomaterials, restoring mitochondrial function has emerged as a promising therapeutic direction (Chen et al., 2019). Smart hydrogels, with programmable physicochemical properties and strong capacity for drug loading and controlled release, provide a suitable platform for this purpose (Chen L. et al., 2024). By exploiting endogenous responsiveness, these materials can deliver drugs or regulatory factors that target mitochondria to the lesion site and dynamically support mitochondrial homeostasis. Such approaches dampen mitochondria linked inflammatory signaling, repair impaired metabolic functions, and reestablish cellular redox balance. By contrast, few studies have improved mitochondrial function through externally stimulated hydrogels. Accordingly, this section focuses on hydrogels that rely on endogenous response mechanisms to regulate mitochondrial function in OA (Table 4).

**TABLE 4:** Endogenous stimuli-responsive hydrogels targeting mitochondrial function.

Endogenous stimuli	Material components	Responsive mechanism	Bioactive agent	Effects	References
pH	Spirulina platensis–derived extracellular vesicles (SP-EVs), rhein (Rh)	the anthraquinone scaffold of Rh	SP-EVs, Rh	alleviate oxidative stress and the resulting mitochondrial dysfunction, promote ATP replenishment, and restore the mitochondrial membrane potential in chondrocytes	Liang et al. (2025)
	OHA, adipic dihydrazide–grafted HA (HA-ADH), Se nanoparticles (SeNPs)	Schiff base bonds between OHA and HA-ADH	SeNPs	sustain GPX1 activation, provide prolonged antioxidant effects, and restore redox homeostasis	Hu et al. (2023a)
ROS	aldehyde-functionalized hyaluronic acid–based hydrogel microspheres (AHAMA-HMs), HA-SeNPs	SeNPs–HA interactions	HA-SeNPs	specifically target chondrocytes, reduce ROS-mediated NF-κB activation, restore expression of mitochondrial electron transport chain complex I and ATP synthase, and improve mitochondrial function	Liu et al. (2025a)
	nicotinamide mononucleotide (NMN), MnO_2_, HA–triphenylphosphine (HA-TPP), HA- poly (L-lysine) (HA-PLL), PNIPAm	MnO_2_ undergoes reductive dissolution	NMN, MnO_2_	enhance the activities of antioxidant enzymes such as SOD, CAT, and GPx, increase the NAD^+^/NADH ratio, maintain mitochondrial metabolic homeostasis, and suppress the release of SASP factors	Shen et al. (2025)
	GelMA, PFC@PLGA/PPS nanoparticles, liposome encapsulated catalase (Lipo-CAT)	thioether groups of PPS	CAT, PFC	by activating the UPR^mt^ pathway mediated by SIRT3, lower ROS levels, regulate mitophagy, and maintain mitochondrial homeostasis	Lu et al. (2025)
	Polyethyleneimine (PEI), TPP, HPB nanozyme, chondrocyte membrane (CM), GelMA, GA	decomposition catalyzed by HPB nanozyme	HPB, CM	eliminate mtROS in inflammatory chondrocytes, reduce mtDNA leakage, inhibit the cGAS–STING–NF-κB signaling pathway, and enhance chondrocyte function	Fan et al. (2025)
Temperature	modified mRNA encoding Atf5 (mod^Atf5^), BMSCs-derived exosomes, PLGA−PEG−PLGA	PLGA−PEG−PLGA	Ex^modAtf5^	activate UPR^mt^ and autophagy in chondrocytes, through regulation of the mTOR/ULK1 signaling pathway mediated by the mitochondrial protease ClpP, enhance their autophagic capacity	Ma et al. (2024)
	F127, insulin-like growth factor I (IGF-I), recombinant adeno-associated virus (rAAV) gene vector, autologous mitochondria	F127	IGF-I, autologous mitochondria	SOX9 and the mitochondrial fusion protein Mfn1 were upregulated, whereas the mitochondrial fission protein Drp1 and proinflammatory factors were downregulated	Zhong et al. (2025)

### 3.1 pH responsive hydrogels

Mitochondrial dysfunction is a key mechanism that drives chondrocyte apoptosis, inflammatory activation, and extracellular matrix degradation. To address this, investigators have employed pH responsive hydrogels containing protonatable or acid labile linkages to deliver mitochondrial regulators directly to diseased chondrocytes. In acidic conditions these carriers release their cargo, restore mitochondrial homeostasis, scavenge reactive oxygen species (ROS), and recalibrate cellular energy metabolism.

**FIGURE 4:**
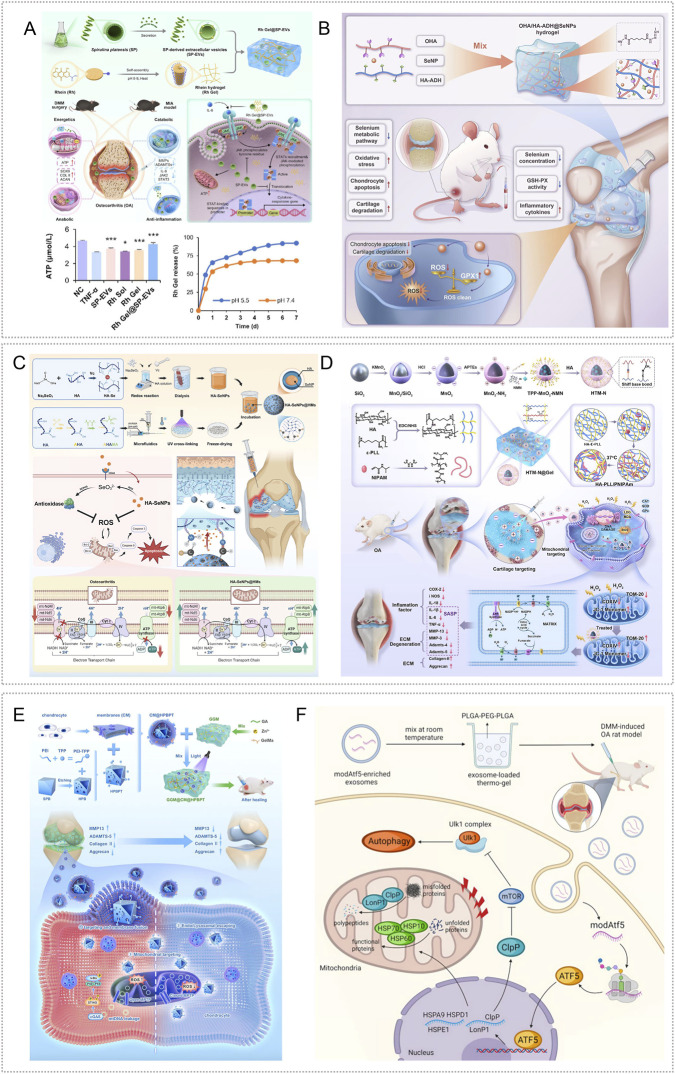
Endogenous stimuli-responsive hydrogel platforms for regulating mitochondrial function. **(A)** Rhein self-assembled hydrogel loaded with Spirulina platensis exosomes (Rh Gel@SP-EVs) shows pH 5.5 responsive release and synergistic anti-inflammatory activity; it restores chondrocyte mitochondrial membrane potential, increases ATP, and inhibits JAK–STAT3. Reproduced with permission from Ref. (Liang et al., 2025), Copyright 2025, American Chemical Society. **(B)** OHA/HA-ADH@SeNPs releases selenium nanoparticles under mild acidity to reinforce mitochondrial glutathione peroxidase defense (activating GPX1), thereby reducing oxidative stress and promoting cartilage repair. Reproduced with permission from Ref. (Hu W. et al., 2023), Copyright 2023, Elsevier. **(C)** HA-SeNPs@AHAMA-HMs releases SeNPs on demand in high ROS conditions; the particles display GPx like activity to remove ROS and, via CD44 mediated delivery, enable nanoscale repair of mitochondrial function. Reproduced with permission from Ref. (Liu J. et al., 2025), Copyright 2025, Elsevier. **(D)** HTM-N@Gel embeds HA-TPP coated NMN@MnO_2_ within an HA–PLL/PNIPAm matrix; ROS responsiveness enhances SOD, CAT, and GPx activities, raises the NAD^+^/NADH ratio, and suppresses SASP, supporting targeted restoration of mitochondrial function. Reproduced with permission from Ref. (Shen et al., 2025), Copyright 2025, Elsevier. **(E)** GGM@CM@HPBPT encapsulates PEI-TPP–modified HPB nanozymes cloaked with chondrocyte membranes; the ROS responsive system targets chondrocytes and their mitochondria, selectively clears mtROS, limits mtDNA leakage, and inhibits cGAS–STING–NF-κB signaling. Reproduced with permission from Ref. (Fan et al., 2025), Copyright 2025, Elsevier. **(F)** Thermosensitive PLGA–PEG–PLGA gel carrying mod^Atf5^ BMSC engineered exosomes (Gel@Ex^modAtf5^) undergoes body temperature gelation with sustained release; it activates UPR^mt^ and enhances autophagy while inhibiting mTOR/ULK1 via ClpP, thereby repairing mitochondrial function. Reproduced with permission from Ref. (Ma et al., 2024), Copyright 2024, American Chemical Society.

Extracellular vesicles derived from Spirulina platensis (SP-EVs) contain antioxidant and ATP-related bioactives as well as metabolism-linked components, making them attractive for OA therapy (Ansari et al., 2023). SP-EVs can modulate intercellular communication and energy balance. To achieve sustained intraarticular delivery, Liang et al. used a rhein self-assembled hydrogel (Rh Gel@SP-EVs) that is pH responsive under mildly acidic conditions and exerts synergistic anti-inflammatory effects. The platform attenuated inflammation-driven oxidative stress, reversed mitochondrial dysfunction, and restored mitochondrial membrane potential in chondrocytes. By improving mitochondrial performance, it replenished ATP and helped maintain the balance between anabolism and catabolism in cartilage matrix, thereby slowing OA progression. In DMM- and MIA-induced mouse models, the system also modulated JAK-STAT3 signaling and suppressed inflammatory responses (Liang et al., 2025) (Figure 4A).

Glutathione peroxidase (GPx) catalyzes the reduction of H2O2 and organic peroxides to water or the corresponding alcohols using glutathione, thereby preserving intracellular redox homeostasis (Chen B. et al., 2024). Enhancing GPx activity, or reinforcing mitochondrial antioxidant defenses with GPx mimetics or nanozymes, is a promising approach for improving mitochondrial function in OA. Hu et al. created an OHA/HA-ADH@SeNPs hydrogel via a one-step method that combined a Schiff base reaction between oxidized hyaluronic acid (OHA) and adipohydrazide-grafted hyaluronic acid (HA-ADH) with uniform dispersion of selenium nanoparticles (SeNPs). The embedded SeNPs were released in a mildly acidic milieu, enabling targeted and timely intervention. In DMM-induced rat OA, OHA/HA-ADH@SeNPs sustained activation of GPX1, produced prolonged antioxidant effects, and reestablished redox balance (Hu W. et al., 2023) (Figure 4B). Most studies leverage joint-cavity acidification for targeted delivery of mitochondrial antioxidants, metabolic modulators, or autophagy-promoting molecules to diseased chondrocytes to restore mitochondrial homeostasis. The goal is not faster release, but aligning the material’s activation window with lesion-site acidity. Release kinetics can be tuned through crosslink density and diffusion pathways to couple dosing with mitochondrial functional recovery in a predictable dose–time relationship. Adding mitochondrial targeting or enhanced cellular uptake can further reduce off-target release and improve homeostasis restoration, shifting from antioxidant supplementation toward redox homeostasis remodeling.

### 3.2 ROS responsive hydrogels

Excess reactive oxygen species (ROS) disrupt the mitochondrial membrane potential and electron transport, resulting in mtDNA damage and impaired respiration; they also activate cell death pathways mediated by mitochondria and exacerbate oxidative stress (Venditti et al., 2013). Scavenging ROS is therefore central to restoring mitochondrial function and slowing OA progression. ROS responsive hydrogels release cargo selectively in regions of high oxidative stress, thereby restoring membrane potential, increasing ATP output, removing excess ROS, and interrupting mitochondrial inflammatory signaling. These effects limit chondrocyte apoptosis, improve energy metabolism, and promote cartilage repair (He et al., 2024).

Selenium nanoparticles (SeNPs) undergo direct redox reactions with ROS, neutralizing them while generating selenite. The resulting SeO_3_^2-^ serves as a precursor for endogenous selenoprotein synthesis and enhances cellular antioxidant capacity (Huang et al., 2003; Manta et al., 2022). Liu et al. created cascade targeted hyaluronic-acid hydrogel microspheres (HA-SeNPs@AHAMA-HMs) by microfluidics, introducing surface aldehydes that formed Schiff-base bonds with amines on damaged cartilage for micron-scale adhesion. HA-SeNPs were co-loaded into the microspheres. Elevated ROS in the OA milieu triggered Se NPs release; reaction with hydrogen peroxide conferred GPx-like activity and removed ROS. The system also exploited CD44 overexpression on OA chondrocytes for nanoscale targeting. In DMM induced rat OA, it reduced ROS driven NF-κB activation, lowered inflammatory cytokines, and restored expression of electron-transport complex I and ATP synthase, thereby improving mitochondrial function and oxidative phosphorylation, limiting apoptosis, and relieving oxidative stress (Liu J. et al., 2025) (Figure 4C).

Chondrocyte senescence sustains synovitis and matrix loss through SASP factors. A key upstream driver is mitochondrial dysfunction: disruption of the respiratory chain reduces ATP, increases electron leakage and ROS, and depletes NAD^+^ (Han et al., 2023; Zhao et al., 2024b). To address this, Shen et al. designed a composite hydrogel that delivers both NAD^+^ precursors and oxygen. Hollow manganese-dioxide nanoparticles loaded with nicotinamide mononucleotide were coated with hyaluronic-acid–triphenylphosphine via Schiff-base chemistry to yield mitochondria targeted particles (HTM-N). Embedding HTM-N in a hyaluronic-acid–polylysine and PNIPAm matrix produced a ROS responsive micro/nano composite (HTM-N@Gel) with cascade targeting to chondrocytes and their mitochondria. *In vitro* and in DMM induced rat OA, the platform responded to ROS, increased activities of superoxide dismutase, catalase, and glutathione peroxidase, raised the NAD^+^/NADH ratio, and reduced SASP factors, thereby maintaining mitochondrial homeostasis and limiting cartilage-matrix degradation (Shen et al., 2025) (Figure 4D). Furthermore, the mitochondrial unfolded-protein response (UPR^mt^) preserves organelle homeostasis by limiting ROS and coordinating mitophagy (Zhou Z. et al., 2022). Lu et al. assembled composite nanoparticles by co-incubating PFC@PLGA/PPS with lipid-encapsulated catalase (CAT-L) and dispersed them in GelMA with LAP photoinitiator; 405-nm irradiation yielded Gel@CAT-L hydrogels. Under high ROS, the material activated SIRT3 mediated UPR^mt^, reduced ROS, regulated mitochondrial autophagy, and improved OA pathology. Analyses of clinical samples showed reduced SIRT3 and UPR^mt^ proteins in OA cartilage (Lu et al., 2025).

To overcome immune clearance, lysosomal trapping, and weak mitochondrial targeting of nanozymes, investigators built a dual targeted system. Polyethyleneimine was combined with triphenylphosphine to produce a positively charged, mitochondria targeted HPB nanozyme (HPBPT), which was then cloaked with a chondrocyte membrane to yield CM@HPBPT for cell specific delivery and lysosome bypass. Loading CM@HPBPT into a gelatin–glycyrrhetinic-acid hydrogel generated GGM@CM@HPBPT. *In vitro* and in ACLT induced mouse OA, this platform improved cell selectivity and immune evasion, accumulated in mitochondria, and under high ROS selectively cleared mitochondrial ROS, reduced mtDNA leakage, inhibited the cGAS/STING/NF-κB pathway, and restored chondrocyte function, thereby dampening inflammation (Fan et al., 2025) (Figure 4E). ROS-responsive hydrogels can deliver modules such as SeNPs, oxygen supply, or nanoenzymes on demand within regions of high oxidative stress. By lowering excessive mitochondrial ROS, they can restore membrane potential and energy metabolism, thereby reducing apoptosis and matrix degradation. Design should align activation thresholds with the local ROS range at the lesion site and tune release kinetics to limit exposure duration, preferentially removing excess ROS while preserving physiological ROS signaling to reestablish redox homeostasis. Further studies are needed to validate improved targeting, long-term safety, and durable efficacy.

### 3.3 Temperature responsive hydrogels

A modest but persistent temperature rise occurs within osteoarthritic lesions, most notably in synovial tissue where inflammation and cellular metabolism are heightened (De Marziani et al., 2025). After intraarticular injection, thermoresponsive hydrogels quickly gel at local lesion temperature and provide sustained delivery of mitochondria-targeted agents. In doing so, they repair mitochondrial damage, restore energy metabolism, remove excess reactive oxygen species (ROS), and interrupt inflammatory cascades, offering a novel therapeutic approach.

Under mitochondrial stress, activating transcription factor 5 (ATF5) upregulates mitochondrial chaperones and proteases in chondrocytes. In parallel, modified mRNA (modRNA) has emerged as an efficient vehicle for nucleic-acid therapeutics (Andries et al., 2015; Deng and Haynes, 2017). Ma et al. combined Atf5-carrying modRNA with bone-marrow mesenchymal stem-cell–derived engineered exosomes to generate Ex^modAtf5^, and used an injectable PLGA–PEG–PLGA hydrogel (Gel) as the carrier to achieve sustained release (Gel@Ex^modAtf5^). In a DMM-induced rat model, controlled release at body temperature activated the mitochondrial unfolded protein response (UPR^mt^) and autophagy in chondrocytes, preserved mitochondrial function, enhanced extracellular-matrix synthesis, and reduced subchondral bone sclerosis. Mechanistically, UPR^mt^ augmented autophagy through regulation of the mTOR/ULK1 pathway via the mitochondrial protease ClpP (Ma et al., 2024) (Figure 4F).

Mitochondrial gene targeting remains challenging in OA because mtDNA haplotypes and mutations are heterogeneous and variable (Liu H. et al., 2023). To address this, investigators used mitochondria as a delivery platform for recombinant adeno-associated virus (rAAV) vectors encoding insulin-like growth factor I (IGF-I). Autologous mitochondria carrying rAAV-IGF-I were encapsulated in an F127 thermosensitive hydrogel for local delivery. This mitochondria/rAAV-IGF-I system improved proliferation, survival, extracellular-matrix synthesis, and mitochondrial function in transplanted chondrocytes, upregulated SOX9 and the fusion protein Mfn-1, and reduced expression of the fission protein Drp-1 and proinflammatory mediators (Zhong et al., 2025). Temperature responsive hydrogels gel under body heat to prolong intra-articular retention and provide sustained release that activates UPR^mt^, autophagy, or anabolic pathways, thereby improving mitochondrial function and matrix metabolism in OA. Translation is limited by modest joint temperature elevation and mitochondrial heterogeneity. Design should match gelation to the lesion-specific temperature window and tune network strength and diffusion to stabilize release, reducing risks of incomplete gelation or uncontrolled dosing. Improved targeting and long-term safety validation remain needed.

## 4 Targeting cartilage damage mechanisms with smart responsive hydrogels

Destruction of articular cartilage and sclerosis of the subchondral bone are core pathological features in most cases of osteoarthritis (OA). Abnormal bone remodeling driven by dysregulated osteoblast and osteoclast activity plays a pivotal role (Hu W. et al., 2021). In early disease, increased trabecular porosity and turnover, reduced bone mineral density, defective mineralization, and disorganization of the matrix are thought to arise from osteochondral crosstalk mediated by enlarged interface pores and vascular invasion. These alterations often coincide with, or precede, early cartilage injury, diminish the shock absorbing capacity of the subchondral plate, and accelerate degeneration of the overlying cartilage (Bolbos et al., 2008; Hügle and Geurts, 2016; Hu Y. et al., 2021). The precise mechanisms by which interactions between cartilage and subchondral bone initiate and sustain OA progression remain unclear and merit further study.

Chondrocytes occupy lacunae within an avascular extracellular matrix. In OA cartilage, a proinflammatory subset expressing IL-1 receptor 1 and TNF receptor 2 expands, and the number of senescent chondrocytes expressing the aging marker P16^INK4A^ increases markedly (Grandi et al., 2020; Sahu et al., 2022). Shifts in chondrocyte subpopulations lead to depletion of hyaluronic acid (HA), proteoglycan 4 (PRG4), and type II collagen (Col II) in hyaline cartilage, reflecting cumulative tissue wear together with an imbalance between anabolism and catabolism. The result is inferior mechanical performance and loss of physiological load bearing capacity (Tan et al., 2022). Morphologically, the normal arcuate arrangement of collagen fibers becomes disrupted and disorganized, compromising structural integrity and accelerating mechanical degradation. During attempted repair, fibrotic change arises in damaged hyaline cartilage as proliferating chondrocytes adopt a fibroblast like phenotype (Santos et al., 2019; Rim and Ju, 2020). Such fibrocartilage lacks the water retention and wear resistance of native hyaline cartilage; although it can temporarily fill defects, it cannot withstand prolonged joint loading and wear. In parallel, chondrocytes sense the physical milieu through mechanoreceptors and ion channels that activate downstream pathways, including MAPK and NF-κB, thereby regulating inflammation, matrix synthesis and degradation, and apoptosis (Hodgkinson et al., 2022).

Among emerging therapeutic strategies, targeted cartilage repair has become a major focus. Stem cell therapy shows promise by secreting growth and immunomodulatory factors that support regeneration. Gene therapy can enhance chondrogenic capacity by delivering prochondrogenic genes such as FGF18 and YAP (Fu et al., 2019; Xiang et al., 2022; Chu et al., 2024). Metabolic modulation and RNA based approaches (siRNA, miRNA, circRNA) further protect cartilage and promote repair by improving cellular energy status or suppressing catabolic mediators (Li et al., 2020; DeJulius et al., 2024). Biomaterial strategies, particularly stimuli-responsive hydrogels, have advanced substantially. These platforms sense endogenous or exogenous cues to enable controlled release of chondrogenic agents, deliver growth factors and genes, and guide stem cell differentiation, underscoring strong translational potential (Enayati et al., 2024; Wu S. et al., 2024).

### 4.1 Endogenous stimuli-responsive hydrogels for targeted cartilage repair in osteoarthritis

Cartilage degeneration arises from persistent mechanical loading, oxidative stress, and matrix proteolysis, which reduce chondrocyte number and secretory capacity and thereby weaken intrinsic repair. In response to the altered joint microenvironment, investigators have developed endogenous stimuli-responsive smart hydrogels that release chondrogenic cues with spatiotemporal precision. These platforms promote stem cell chondrogenic differentiation or restore chondrocyte metabolism, reconstructing cartilage architecture and advancing regenerative therapy for osteoarthritis (Table 5).

**TABLE 5:** Endogenous stimuli-responsive hydrogels targeting cartilage damage.

Endogenous stimuli	Material components	Responsive mechanism	Bioactive agent	Effects	References
pH	Sirt1-encoding saRNA, polylysine-modified H ferritin hollow nanocages (4LF), chitosan, OCS, β-GP	disassembly of the 4LF	saRNA	upregulate Sirt1 protein expression, activate PI3K/Akt signaling, inhibit TLR4/NF-κB p65 signaling, reduce chondrocyte apoptosis, and promote cell migration	Xu et al. (2025a)
	DSPE-Hyd-PEG, WYRGRL, cationic lipid DOTAP, KGN, GelMA	the hydrazone linkage in DSPE–Hyd–PEG	WYRGRL, KGN	promote chondrogenesis, enhance anabolic activity, and suppress catabolic activity; induce chondrogenic differentiation by facilitating collagen trimer assembly and the synthesis of collagen-related extracellular matrix	Li et al. (2025e)
	MnO_2_, bovine serum albumin (BSA), HA, platelet-rich plasma (PRP)	Schiff base bond	MnO_2_, PRP	alleviate severe oxidative stress and promote chondrocyte proliferation	Zhou et al. (2022a)
ROS	CeO_2_, Fe_2_O_3_, thiol crosslinked collagen scaffold (CSH), poly (lactic-co-glycolic acid) (PLGA)	Fe_2_O_3_, CeO_2_ respond to elevated ROS levels	Fe_2_O_3_, CeO_2_	rapidly scavenge ROS and relieve inflammation in the early stage; sustain release in the later disease phase to further promote cartilage repair	Chen et al. (2024e)
	ROS responsive copolyme (PEG-PCL-TSPBA), KGN, dexamethasone (Dex), WYRGRL, GelMA	PEG-PCL-TSPBA	KGN, Dex, WYRGRL	exhibits cartilage targeting, scavenges excess ROS, locally releases Dex and KGN, and promotes stem cell differentiation into chondrocytes	Yu et al. (2022)
	tannic acid (TA), ZIF-8 MOFs, parathyroid hormone related peptide 1 (PTHrP-1), phenylboronic-acid modified gelatin hydrogel (GP)	boronate ester bonds between the GP hydrogel and TA-ZIF@P1	TA, PTHrP-1	resist cellular injury induced by ROS and IL-1β; transcriptomic profiling indicates enhanced chondrocyte proliferation via inhibition of the Wnt/β-catenin pathway and activation of PI3K/AKT signaling	Shi et al. (2025)
Enzyme	streamlined ZnO nanoparticles, miR-17-5p (miR-17), GelMA	the gelatin peptide chains of GelMA were hydrolyzed	miR-17, Zn^2+^	activate the Ihh/PTHrP signaling pathway to recruit BMSCs and drive chondrogenic differentiation; target MMP13 and ADAMTS5 to inhibit ECM degradation	Li et al. (2025c)
	HAMA, SKP peptide, K peptide, Q peptide, MMP-13 sensitive peptide, collagen targeting peptide (C5-24), sulfated chitosan (SCS)	MMP-13 sensitive peptide	SKP peptid, SCS	promote recruitment of SMSCs and their chondrogenic differentiation, enhance secretion of reparative factors, suppress the tendency of SMSCs to differentiate into fibroblasts, and downregulate Ihh and type I collagen expression	Chen et al. (2025c)
	proteoglycans (PGs), poly (L-lysine)–glucono-δ-lactone (PLL-glu, Pg), oxidized dextran (OD)	PLL can be cleaved by proteases	Pg, OD	promote chondrocyte proliferation, adhesion, and migration; enhance ECM deposition; and upregulate cartilage specific markers, including Col-II, aggrecan, and sGAG	Hu et al. (2023b)
Temperature	imidazolidinyl urea (IU), carbamate linkage, 4, 4′-methylenediphenyl diisocyanate (MDI), PEG, TA, KGN	multiple hydrogen bonding and polyurethane microphase separation	TA, KGN	modulate inflammatory responses, scavenge excess ROS, and inhibit bacterial adhesion, thereby establishing a favorable microenvironment for MSC homing and subsequently inducing their differentiation into chondrocytes	Yang et al. (2023)
	circRNA3503-enriched exosomes derived from SMSCs (circRNA3503-OE-sEVs), triblock copolymer hydrogel (PDLLA-PEG-PDLLA, PLEL)	PLEL	circRNA3503-OE-sEVs	act as a ceRNA to regulate SOX9 expression and promote ECM synthesis; it can also mediate downstream regulation by sponging hsa-miR-181c-3p and hsa-let-7b-3p, thereby suppressing MMP-13 and ADAMTS-5 expression	Tao et al. (2021)
	Heparin (Hep), ε-poly (L-lysine) (EPL), platelet lysate (PL), PLEL	PLEL	PL	slow cartilage degeneration at early stages and promote cartilage repair at later stages. RNA sequencing indicates that the protective effect of PL is closely associated with its regulation of HAS1 expression	Tang et al. (2021)

#### 4.1.1 pH responsive hydrogels

**FIGURE 5:**
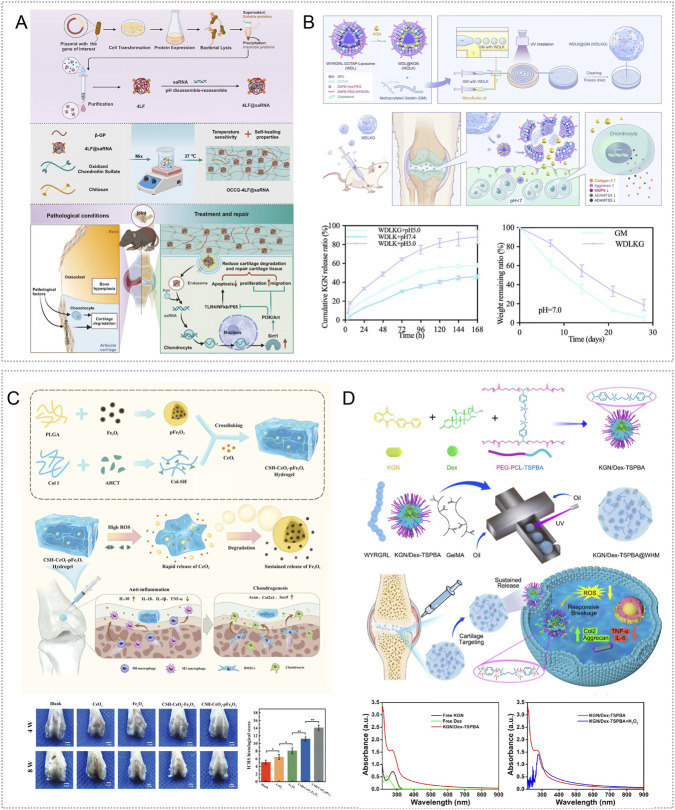
pH and ROS responsive hydrogels targeting cartilage injury. **(A)** OCCG-4LF@saRNA: a chitosan/oxidized chondroitin sulfate/β-glycerophosphate matrix loaded with 4LF@saRNA. The platform shows pH responsiveness and lysosomal escape, targets chondrocytes to upregulate Sirt1, activate PI3K/Akt, inhibit TLR4/NF-κB p65, and promote cartilage repair. Reproduced with permission from Ref. (Xu L. et al., 2025), Copyright 2025, Elsevier. **(B)** WDLKG composite system: DSPE–Hyd–PEG/DOTAP liposomes carrying kartogenin (KGN), surface grafted with the type II collagen–binding peptide WYRGRL and rendered pH responsive via the hydrazone linkage (HyD); liposomes are embedded in GelMA for targeted cartilage delivery and sustained release to drive chondrogenesis. Reproduced with permission from Ref. (Li et al., 2025e), Copyright 2025, Elsevier. **(C)** CSH-CeO_2_-pFe_2_O_3_ hydrogel: a thiol crosslinked collagen scaffold (CSH) containing CeO_2_-pFe_2_O_3_ microspheres. Early high ROS triggers rapid CeO_2_ release to quell oxidative stress and inflammation, whereas later sustained Fe_2_O_3_ release enhances cartilage regeneration. Reproduced with permission from Ref. (Chen X. et al., 2024), Copyright 2024, Wiley-VCH GmbH. **(D)** KGN/Dex-TSPBA@WHMs: ROS responsive nanoparticles (PEG–PCL–TSPBA loaded with KGN and dexamethasone) incorporated into WYRGRL-functionalized hydrogel microspheres. The construct targets cartilage, improves cellular uptake, scavenges excess ROS, and promotes stem-cell chondrogenic differentiation. Reproduced with permission from Ref. (Yu et al., 2022), Copyright 2022, American Chemical Society.

Small activating RNA (saRNA) is a 21-nucleotide double-stranded RNA that binds Ago2 and targets promoter regions to activate transcription and increase protein expression, but it faces challenges similar to siRNA in stability, membrane permeability, and delivery efficiency (Portnoy et al., 2016; Kingwell, 2021). Xu et al. addressed these barriers by introducing four lysine residues at the N terminus of the FTH gene to generate polylysine-modified H ferritin hollow nanocages (4LF) as a carrier for Sirt1 saRNA. The resulting 4LF@saRNA efficiently delivered saRNA to chondrocytes; lysine decoration conferred a proton sponge effect that protected cargo from lysosomal degradation, while pH-triggered disassembly of the 4LF shell released saRNA into the cytoplasm to upregulate Sirt1. The nanoparticles were then incorporated into a thermosensitive self-healing hydrogel composed of chitosan, oxidized chondroitin sulfate (OCS), and sodium beta glycerophosphate (βGP), yielding the OCCG-4LF@saRNA system. *In vitro* and in DMM-induced mouse OA, this platform increased Sirt1 protein, activated PI3K/Akt signaling, and suppressed TLR4/NF-κB p65, thereby reducing chondrocyte apoptosis and promoting migration (Xu L. et al., 2025) (Figure 5A).

Kartogenin (KGN), a chondrogenic small molecule, binds filamin A and blocks its interaction with core-binding factor β (CBFβ), thereby activating CBFβ and upregulating RUNX1 to drive chondrocyte differentiation and chondroprotection (Johnson et al., 2012). The hexapeptide WYRGRL targets type II collagen, and PEGylation prolongs its retention in cartilage [83]. Building on these features, Li and colleagues created a cartilage-targeted, pH responsive liposomal system composed of DSPE-Hyd-PEG, surface-modified with WYRGRL and containing the cationic lipid DOTAP, with KGN as payload. The liposomes were embedded in methacrylated gelatin (GelMA) microspheres to form WDLKG hydrogel microspheres. WDLKG enhanced anabolic activity, suppressed catabolic processes, and promoted chondrogenesis; transcriptomics indicated primary actions on collagen trimer formation and collagen-associated extracellular matrix synthesis. In DMM-induced mouse OA, the system provided sustained release and inhibited cartilage degeneration (Li et al., 2025e) (Figure 5B).

Platelet-rich plasma (PRP), which contains abundant platelets, growth factors, and cytokines, is widely used for cartilage repair (Amiri et al., 2023). Investigators dispersed bovine-serum-albumin–manganese-oxide (BSA–MnO_2_) nanozymes within a hyaluronic-acid/PRP gel crosslinked via Schiff-base chemistry, producing an acid responsive hydrogel. In acidic conditions the matrix released both BM nanoparticles and PRP-derived factors: the nanozymes mitigated oxidative stress, and PRP components promoted chondrocyte proliferation. *In vitro* and in MIA-induced rat OA, this platform slowed disease progression through combined mechanical dissipation, anti-inflammatory effects, and enhanced cartilage repair (Zhou T. et al., 2022). pH-responsive hydrogels enable targeted delivery and controlled release of active agents such as saRNA, KGN, and PRP to promote chondrogenic differentiation and slow degeneration in osteoarthritis cartilage repair. Effective design requires matching the pH activation window to the extent of joint acidification, tuning release timing through crosslink strength and diffusion channels, and enhancing cartilage affinity or cellular uptake to limit unproductive diffusion while improving deep penetration and repair efficiency.

#### 4.1.2 ROS responsive hydrogels

In addition to incorporating oxidation sensitive groups such as thio-ketones and thioethers to build ROS responsive hydrogels, antioxidant nanomaterials—including MnO_2_, CeO_2_, and Prussian blue—can be integrated into hydrogel matrices (Xu et al., 2022; Liu J. et al., 2023). These platforms remove excess ROS, dampen inflammation, and limit cartilage catabolism, making them attractive for bone and cartilage tissue engineering.

Repair of OA lesions proceeds through inflammatory and proliferative phases, yet the lack of phase specific therapy can shorten treatment durability (Bohaud et al., 2021). To address this, investigators created a composite hydrogel that combines CeO_2_ nanoparticles with Fe_2_O_3_-loaded poly (lactic-co-glycolic acid) (PLGA) microspheres. Collagen scaffolds were thiol crosslinked and embedded with CeO_2_ and PLGA–Fe_2_O_3_ to yield a collagen–CeO_2_–PLGA microsphere–Fe_2_O_3_ hydrogel (CSH-CeO_2_-pFe_2_O_3_). The material supported cell adhesion, proliferation, and chondrogenic differentiation. Under early high-ROS conditions, it rapidly released CeO_2_ to neutralize oxidative stress and reduce inflammation; during the later regenerative phase, Fe_2_O_3_ was released in a sustained manner to further enhance cartilage repair. In a rat cartilage-defect model, the hydrogel lowered inflammation and improved regeneration, achieving favorable ICRS macroscopic scores and illustrating its potential for staged OA therapy (Chen X. et al., 2024) (Figure 5C).

Targeted, ROS responsive microspheres have also been developed for cartilage delivery. Researchers first prepared ROS responsive nanoparticles (KGN/Dex-TSPBA) by electrostatically assembling a TSPBA-containing PEG-PCL copolymer with kartogenin (KGN) and dexamethasone (Dex). They then used microfluidics and UV crosslinking to generate monodisperse, injectable hydrogel microspheres (KGN/Dex-TSPBA@WHMs) functionalized with the type II collagen–targeting peptide WYRGRL. In MIA-induced rat OA, these chondro-targeted microspheres showed high intraarticular retention and enhanced cellular uptake. Reaction of the nanoparticles with ROS induced depolymerization that both scavenged ROS and triggered local release of Dex and KGN at the lesion, promoting stem-cell chondrogenesis and reducing inflammation (Yu et al., 2022) (Figure 5D).

Parathyroid hormone–related peptide 1 (PTHrP-1) promotes chondrocyte proliferation and differentiation and thus has therapeutic value for cartilage repair (Huang et al., 2020). A tannic-acid functionalized ZIF-8 carrier (TA-ZIF) was loaded with PTHrP-1 (TA-ZIF@P1) and combined with a boronic-acid modified gelatin hydrogel (GP) to form a GPTP hydrogel (GP@TA-ZIF@P1). Phenolic hydroxyls on TA-ZIF@P1 formed dynamically reversible borate bonds with the GP network, enabling ROS responsive release. *In vitro*, GPTP enhanced chondrocyte proliferation and protected against ROS- and IL-1β-induced injury. Transcriptomics indicated inhibition of Wnt/β-catenin and activation of PI3K/AKT signaling as key mechanisms. In DMM-induced murine OA, GPTP reduced perichondrial remodeling, preserved cartilage, and promoted repair (Shi et al., 2025). ROS-responsive hydrogels can preferentially remove excess reactive oxygen species, providing anti-inflammatory activity, limiting matrix degradation, and promoting chondrogenic differentiation. They can be paired with antioxidant nanomaterials and peptides such as KGN, Dex, and PTHrP-related peptide 1 to support staged therapy. Design should match activation thresholds to lesion-specific ROS levels and tune release timing through network density and diffusion pathways. This reduces premature release outside diseased sites while preserving physiological ROS signaling. Future work should improve system stability and targeting efficiency, and further clarify ROS-responsive mechanisms and downstream signaling regulation.

#### 4.1.3 Enzyme responsive hydrogels

**FIGURE 6:**
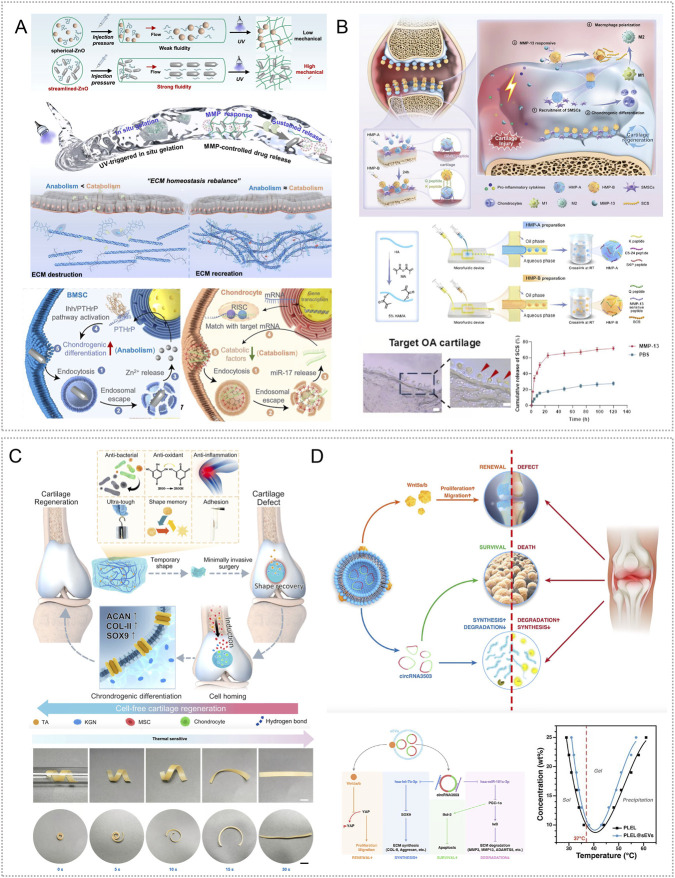
Enzyme responsive and thermosensitive hydrogels targeting cartilage injury. **(A)** strZPM Gel (GelMA matrix) loaded with streamlined ZnO@PEI/miR-17 nanocomposites; MMP responsive. Released Zn^2+^ activates the Ihh/PTHrP axis to recruit BMSCs and drive chondrogenesis, while miR-17 suppresses MMP13 and ADAMTS5 to limit ECM degradation. Reproduced with permission from Ref. (Li W. et al., 2025), Copyright 2025, Springer Nature. **(B)** dmHMPs dual module: HMP-A localizes to cartilage via the C5-24 peptide and induces SMSC chondrogenesis; HMP-B docks to HMP-A through K/Q peptide pairing and, upon MMP-13 cleavage, releases sulfated chitosan to promote macrophage polarization and reduce fibrosis, achieving full-thickness cartilage regeneration. Reproduced with permission from Ref. (Chen X. et al., 2025), Copyright 2025, Wiley-VCH GmbH. **(C)** PTK hydrogel: a polyurethane (PMI) platform co-loaded with tannic acid and KGN. TA imparts tissue adhesion, and its multivalent hydrogen-bond network enables rapid shape recovery at 37 °C for conformal defect filling; KGN complements TA by reducing inflammation and ROS, guiding BMSC homing, and promoting chondrogenesis to realize full-thickness repair. Reproduced with permission from Ref. (Yang et al., 2023), Copyright 2023, Springer Nature. **(D)** PLEL@circRNA3503-OE-sEVs: a PLEL triblock copolymer hydrogel encapsulating SMSC-derived exosomes enriched in circRNA3503; gels rapidly at body temperature. circRNA3503 acts as a ceRNA to upregulate SOX9 and, by sponging hsa-miR-181c-3p and hsa-let-7b-3p, restores ECM homeostasis. Reproduced with permission from Ref. (Tao et al., 2021), Copyright 2021, Elsevier.

Zn^2+^ act as cofactors for many enzymes involved in DNA synthesis, immune regulation, and bone metabolism, and therefore have therapeutic potential (Read et al., 2019). As a member of the miR-17–92 cluster, miR-17-5p (miR-17) suppresses key matrix-degrading mediators, including MMP13 and ADAMTS5, and downregulates additional metalloproteinases to resist extracellular matrix (ECM) loss (Ventura et al., 2008). Li et al. co-delivered miR-17 with streamlined ZnO nanoparticles to cartilage defects to rebalance ECM synthesis and degradation and achieve steady-state repair. The GelMA/str-ZnO@PEI/miR-17 system (strZPM Gel) reproduced multiple biophysical features of native ECM and supported robust cell proliferation, migration, and adhesion with favorable biocompatibility and degradability. This MMP responsive platform provided sustained release after intraarticular injection in rat OA cartilage defects. Released Zn^2+^ activated the Ihh/PTHrP pathway, promoting recruitment and chondrogenic differentiation of bone marrow mesenchymal stem cells, while miR-17 targeted catabolic factors such as MMP13 and ADAMTS5 to curb ECM breakdown (Li W. et al., 2025) (Figure 6A).

The synovium contributes to cartilage repair by recruiting synovial-derived mesenchymal stem cells (SMSCs), which can differentiate into chondrocytes and secrete matrix (McGonagle et al., 2017). To enable full thickness regeneration, Chen et al. designed a dual-module system (dmHMPs) comprising HMP-A and HMP-B. HMP-A localized to damaged cartilage via the collagen-targeting peptide C5-24 and initiated repair by recruiting SMSCs and driving their chondrogenic differentiation through the SKPPGTSS peptide. HMP-B anchored onto HMP-A via K-peptide–Q-peptide interactions and responded rapidly to MMP-13 at injury sites to release sulfated chitosan, which induced macrophage polarization and enhanced secretion of reparative factors. This immune modulation limited fibroblastic drift of SMSCs and improved repair quality. In DMM-induced OA, dmHMPs downregulated Indian hedgehog (Ihh) and the fibrocartilage marker type I collagen, promoting full thickness articular cartilage regeneration (Chen X. et al., 2025) (Figure 6B).

Proteoglycans are major ECM constituents, and their loss accompanies irreversible cartilage degeneration (Wei et al., 2021). Based on structural simulation of proteoglycans, a glycopeptide POD hydrogel was created using polylysine glucono delta lactone (PLL-glu, Pg) as the core protein and oxidized dextran as the polysaccharide component, cross linked via a Schiff base reaction. Among several formulations, Gel-3 (4% PGs and 8% oxidized dextran) performed best. The hydrogel underwent enzyme responsive degradation that facilitated controlled remodeling of type II collagen, supported chondrocyte proliferation, adhesion, and migration, and reduced intracellular ROS. In rabbit OA cartilage defects, it enhanced ECM deposition and upregulated chondrogenic markers including type II collagen, aggrecan, and sulfated glycosaminoglycans (Hu Y. et al., 2023). Enzyme responsive hydrogels enable on-demand drug release within enzyme-rich osteoarthritic lesions to achieve site-specific therapy. By undergoing selective cleavage, they promote MSC differentiation, limit matrix degradation, modulate immune responses, and stimulate cartilage regeneration. When combined with strategies such as miRNA delivery and zinc-ion regulation, their functionality and adaptability are further enhanced. Overall, enzyme-responsive systems share a common principle: they translate elevated enzyme activity at lesion sites into cues for localized release and structural remodeling, enabling a single platform to coordinate anti-degradation, immune regulation, and pro-regeneration processes.

#### 4.1.4 Temperature responsive hydrogels

Temperature responsive hydrogels typically crosslink under physiological conditions through physical interactions without additional crosslinkers. They can be injected to fill cartilage defects *in situ*, form three-dimensional networks that match tissue architecture, and support chondrocyte adhesion, growth, and differentiation, thereby creating an optimal niche for regeneration. Scaffold designs that incorporate “cell homing” cues to recruit endogenous mesenchymal stem cells (MSCs) from trabecular regions have been proposed, an approach that avoids harvesting autologous cartilage and reduces the risk of immune rejection (Vainieri et al., 2020). Yang et al. first introduced imidazolidinyl urea (IU) as a hydrogen-bond enhancer into a polyurethane backbone and, via an addition reaction with PEG and methylene diphenyl 4,4′-diisocyanate (MDI), prepared a polymeric hydrogel (PMI) with strengthened hydrogen bonding. Co-loading tannic acid (TA) and kartogenin (KGN) into PMI produced the final hydrogel (PTK). TA endowed PTK with strong tissue adhesion for stable *in situ* fixation. The material recovered its original shape within 30 s at body temperature, enabling rapid structural recovery after minimally invasive injection and conformal unfolding to the defect surface. *In vitro* and *in vivo* studies showed that PTK modulated inflammation, scavenged excess reactive oxygen species (ROS), and limited bacterial adhesion, thereby establishing a favorable microenvironment for MSC homing. In a rat osteoarthritis (OA) cartilage-defect model, gradual degradation sustained KGN release, promoted bone-marrow MSC migration into the scaffold, induced chondrogenic differentiation, and achieved full-thickness cartilage regeneration (Yang et al., 2023) (Figure 6C).

Circular RNAs regulate chondrocyte apoptosis by modulating microRNAs or downstream targets; their covalently closed structure confers exceptional stability and a key role in cartilage homeostasis (Zhou et al., 2019; Zhang et al., 2021). Small extracellular vesicles (sEVs) from synovial MSCs (SMSCs) can penetrate cartilage, enter chondrocytes, and help maintain extracellular-matrix (ECM) balance, making them attractive carriers for nucleic-acid therapeutics in OA (Tao et al., 2017). Using RNA sequencing, Tao et al. identified circRNA3503 as markedly upregulated during melatonin-induced cellular quiescence. They generated circRNA3503-overexpressing sEVs (circRNA3503-OE-sEVs) from SMSCs and embedded them in a triblock copolymer hydrogel (PDLLA-PEG-PDLLA, PLEL) to form PLEL@circRNA3503-OE-sEVs. After intraarticular injection, the formulation gelled rapidly at 37 °C to create a local scaffold. circRNA3503 reduced IL-1β-induced chondrocyte apoptosis, promoted ECM synthesis, and stabilized the chondrocyte phenotype via SOX9 regulation as a competing endogenous RNA. In a rabbit OA defect model, circRNA3503 also restored ECM homeostasis and suppressed MMP-13 and ADAMTS-5 by adsorbing hsa-miR-181c-3p and hsa-let-7b-3p (Tao et al., 2021) (Figure 6D).

In addition, Tang et al. improved platelet-derived growth factor (PDGF) dispersibility by constructing heparin/ε-polylysine nanoparticles through electrostatic self-assembly. Loading these PDGF-NPs into an ε-polylysine hydrogel yielded a temperature-responsive depot with sustained PDGF release. The system suppressed IL-1β-induced chondrocyte inflammation and dedifferentiation. In an ACLT mouse model, it slowed early cartilage degeneration and promoted repair at later stages. Transcriptomic analysis suggested that protection was associated with regulation of hyaluronan synthase-1 (HAS1) expression (Tang et al., 2021). Temperature responsive hydrogels gel *in situ* at body temperature to prolong intra-articular retention and enable sustained release of chemokines, small-molecule drugs, or circRNAs that suppress inflammation, reduce apoptosis, stabilize the ECM, and support repair. Incorporation of exosomes or cytokines can further enhance homing and phenotypic maintenance of endogenous MSCs. Overall, the approach integrates rapid gelation for local retention with controlled release to dampen inflammation, facilitate MSC recruitment, and subsequently promote chondrogenic differentiation and matrix deposition.

### 4.2 Exogenous stimuli-responsive hydrogels for targeted cartilage repair in osteoarthritis

Exogenous stimuli-responsive hydrogels can undergo structural transitions or release therapeutic payloads in response to exogenous cues, thereby activating chondrogenic programs and promoting cartilage regeneration and reconstruction. With good controllability and biocompatibility, they permit stage specific, dynamic intervention during cartilage repair and are central to precision, adjustable strategies for osteoarthritis (OA) therapy (Table 6).

**TABLE 6:** Exogenous stimuli-responsive hydrogels targeting cartilage damage.

Exogenous stimuli	Material components	Responsive mechanism	Bioactive agent	Effects	References
Light	HA, triethylene glycol (TEG), coumarin	coumarin groups crosslink under UV irradiation	coumarin, HA	improve the quality of early cartilage repair, resulting in higher overall macroscopic scores and superior histologic cartilage repair	Gao et al. (2023)
	SKP peptide, liposomes (Lipo), KGN, AHAMA	the methacrylate groups in AHAMA	KGN, SKP	induce homing of endogenous BMSCs to the lesion and their chondrogenic differentiation; transcriptomic analysis indicates that this process relies on activation of TGF-β signaling via Smad4	Wang et al. (2025a)
Electro	barium titanate nanoparticles, HAMA, polydopamine (pDA), stem cell recruitment peptide (CR)	barium titanate nanoparticles generate localized electric fields	CR, barium titanate nanoparticles	by triggering Ca^2+^ influx to activate the p38 MAPK signaling pathway, thereby promoting cartilage regeneration and enhancing BMSC migration and chondrogenic differentiation	Han et al. (2025)
	collagen matrix, short electrospun poly-L-lactic acid nanofibers (NF-sPLLA)	piezoelectric effect of NF-sPLLA	NF-sPLLA	enhance cell migration, induce stem cells to secrete TGF-β1, promote chondrogenesis, and improve hyaline cartilage architecture	Vinikoor et al. (2023)
	TGF-β1, Silk protein solution, AAM, PLA-PEG-PLA, N-(3-aminopropyl) Methacrylamide (APM)	silk protein chains migrate directionally in response to an applied electric field	n(TGF-β1), silk protein chains	Direct BMSCs toward lineage-specific differentiation, promote hyaline-like cartilage formation, and improve integration with the surrounding host tissue	Wang et al. (2025e)
Magnetic	Fe_3_O_4_NPs, GMHA, hydroxyapatite (HAp), VEGF	hollow porous magnetic microspheres of Fe_3_O_4_NPs	Fe_3_O_4_NPs, VEGF	facilitate efficient loading and release of exogenous growth factors such as VEGF, enhance osteogenic differentiation of BMSCs, and promote new bone formation	Rong et al. (2024)
Mechanical	HAMA, sgFGF18-loaded liposomes (Lipo), HEK293 cell–derived exosomes (CAP/EXO)	hybrid exosomes formed by membrane fusion between exosomes and liposomes	CAP/FGF18-hyEXO	provide sustained lubrication and, by modulating the PI3K/AKT signaling pathway, promote chondrocyte proliferation; transcriptomic analysis reveals synergy between *in vivo* FGF18 activation and the lubrication function	Chen et al. (2024d)
	Rapamycin (RAPA), HSPC liposomes, HAMA	incorporation of liposomes into HAMA	RAPA	target the negatively charged cartilage surface, form a durable boundary lubrication layer, and release rapamycin (RAPA) to clear excess ROS and suppress chondrocyte apoptosis, thereby maintaining cellular homeostasis through enhanced autophagy	Lei et al. (2022)

**FIGURE 7:**
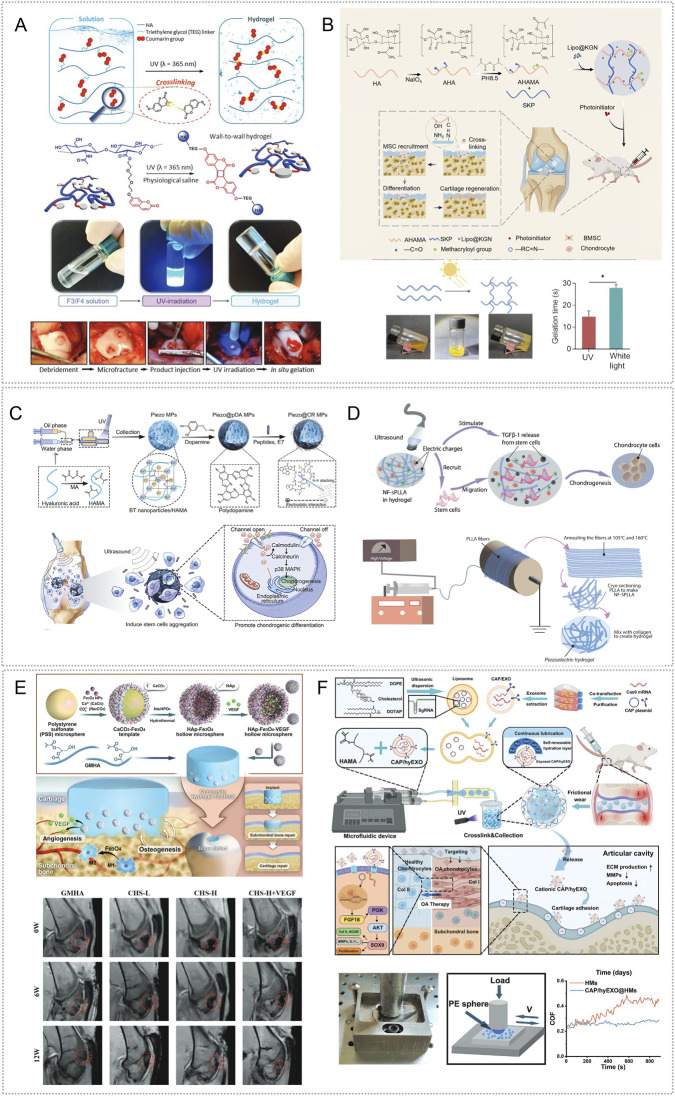
Exogenous stimuli-responsive hydrogels targeting cartilage injury. **(A)** Light responsive HA–TEG–coumarin hydrogel synthesized from HA grafted with TEG–coumarin; rapid *in situ* UV photocrosslinking precisely fills microfracture pores and, combined with microfracture, improves histologic repair. Reproduced with permission from Ref. (Gao et al., 2023), Copyright 2023, Wiley-VCH GmbH. **(B)** Photoresponsive hydrogel based on aldehyde functionalized AHAMA, loaded with KGN liposomes (Lipo@KGN) and covalently grafted with the SKP peptide; crosslinked *in situ* under 520 nm light, it adheres strongly to cartilage and provides sustained release, inducing endogenous BMSCs to undergo chondrogenic differentiation. Reproduced with permission from Ref. (Wang D. et al., 2025), Copyright 2025, Elsevier. **(C)** Piezo@CR hydrogel microbeads with a BaTiO_3_ nanoparticle–HAMA core, coated with polydopamine and the CR peptide; ultrasound generated electrical cues trigger Ca^2+^ influx, activate p38 MAPK, recruit BMSCs, and promote chondrogenic differentiation. Reproduced with permission from Ref. (Han et al., 2025), Copyright 2025, Wiley-VCH GmbH. **(D)** Piezoelectric hydrogel composed of NF-sPLLA embedded in a collagen matrix; ultrasound activation yields localized electrical signals that induce stem cells to secrete TGF-β1, promote chondrogenesis, and enhance trabecular formation. Reproduced with permission from Ref. (Vinikoor et al., 2023), Copyright 2023, Springer Nature. **(E)** CHS scaffold with a GMHA photocrosslinked matrix embedding hollow porous HAp/Fe_3_O_4_ magnetic microspheres; enables VEGF loading and sustained release (CHS-H-VEGF), shows superparamagnetism and MRI traceability, enhances BMSC osteogenic differentiation, and supports targeted repair with real time monitoring. Reproduced with permission from Ref. (Rong et al., 2024), Copyright 2024, Wiley-VCH GmbH. **(F)** Mechanically responsive HAMA hydrogel encapsulating hybrid exosomes (CAP/FGF18-hyEXO); mechanical stimulation forms a self-renewing hydration layer while CAP guides chondrocyte orientation. *In vivo*, FGF18 is activated and promotes chondrocyte proliferation and ECM synthesis through the PI3K/AKT pathway. Reproduced with permission from Ref. (Chen M. et al., 2024), Copyright 2024, Wiley-VCH GmbH.

Injectable photopolymerizable hydrogels combine *in situ* delivery with minimally invasive fixation and are a leading material class in cartilage regeneration research (Rapp and DeForest, 2020). Although microfracture (MFX) recruits mesenchymal stem cells (MSCs) to defects and promotes fibrocartilage repair, its efficacy wanes over time (Pałka et al., 2025). To enhance outcomes, investigators chemically introduced TEG–coumarin groups into hyaluronic acid (HA) to create a light responsive HA-TEG–coumarin precursor. After injection into defects such as microfracture holes, *in vivo* UV irradiation induced photopolymerization, resulting in an *in-situ* gel with a stable network. In a miniature pig OA defect model, MFX combined with the photocrosslinked HA hydrogel (F3, 30% substitution, 30 mg mL^-1^) outperformed MFX alone and untreated controls, yielding higher macroscopic scores and superior histologic repair (Gao et al., 2023) (Figure 7A). In related work, liposome loaded kartogenin (Lipo@KGN) was covalently linked with the cationic functional peptide SKP onto an aldehyde modified methacrylated HA matrix (AHAMA). In a rat OA defect model, brief white light irradiation at 520 nm produced rapid crosslinking and strong adhesion to cartilage. The hydrogel sustained release of KGN and SKP, induced homing of endogenous bone marrow MSCs, and promoted their chondrogenic differentiation. Transcriptomics indicated reliance on Smad4 mediated activation of TGF-β signaling, and *in vivo* studies showed robust hyaline cartilage regeneration within 8 weeks (Wang D. et al., 2025) (Figure 7B).

Electrical stimulation is another exogenous modality with precise spatiotemporal control. Electrical phenomena are intrinsic to joint physiology (Wu et al., 2022); negatively charged proteoglycans in cartilage generate diffusion, streaming, and osmotic potentials under load, and exogenous electric fields can mimic these cues to promote matrix synthesis and repair (Zhou et al., 2023). Electro responsive hydrogels often incorporate conductive polymers, carbon materials such as graphene or carbon nanotubes, metallic nanoparticles, or bio-ionic liquids (Arif et al., 2025). Han et al. created force-to-electricity hydrogel microspheres (Piezo@CR MPs) by embedding barium titanate nanoparticles in HAMA microspheres and coating them with a polydopamine layer that binds a stem cell recruitment peptide (CR). The system converted external pressure, including ultrasound, into a ∼451 mV signal that enhanced bone marrow MSC migration and chondrogenic differentiation *in vitro*. In rabbit OA defects it generated cartilage-like tissue, delayed degeneration, improved locomotor function, and acted through Ca^2+^ influx and p38 MAPK activation (Han et al., 2025) (Figure 7C). Similarly, short electrospun poly-L-lactic acid nanofibers embedded in collagen produced a piezoelectric hydrogel that generated local electrical signals under ultrasound, stimulated cell migration, induced stem cells to secrete TGF-β1, and promoted chondrogenesis. In a rabbit OA model, ultrasound-activated gel enhanced trabecular bone formation and restored hyaline cartilage structure with mechanical properties approaching healthy tissue (Vinikoor et al., 2023) (Figure 7D). Wang et al. developed a dual-gradient silk-based hydrogel (GS) for osteochondral repair. TGF-β1 was first encapsulated *in situ* using charged monomers to generate nanocapsules, n(TGF-β1). These nanocapsules were then incorporated into a silk solution and enzymatically crosslinked to form the hydrogel. Upon application of an external electric field, silk protein chains migrated to establish a mechanical gradient, with a softer region near the cathode and a stiffer region near the anode. In parallel, the charged hydrogel network and the nanocapsules aligned and redistributed along the electric field, producing a coupled gradient that integrates biomechanical and biochemical cues. *In vitro* studies showed that this material directs lineage-specific differentiation of bone marrow mesenchymal stem cells, with stiffer regions promoting osteogenesis and softer regions enhancing chondrogenesis. In a rabbit full-thickness osteochondral defect model, the dual-gradient hydrogel supported the formation of hyaline-like cartilage and improved integration with surrounding native tissue (Wang Y. et al., 2025).

Superparamagnetic Fe_3_O_4_ nanoparticles (Fe_3_O_4_NPs) are established MRI contrast agents (Yue et al., 2024). Encapsulation of Fe_3_O_4_NPs within hydrogels yields nanomagnetic “ferrogels” with broad biomedical applications (Babaniamansour et al., 2022). Rong et al. used a photopolymerizable GMHA matrix loaded with hollow porous magnetic microspheres made of hydroxyapatite and Fe_3_O_4_NPs. Light induced polymerization secured the microspheres within the network, forming superparamagnetic composite hydrogel scaffolds (CHS). Formulations containing 2 mg mL^-1^ and 10 mg mL^-1^ HAp–Fe_3_O_4_ were designated CHS-L and CHS-H, and a VEGF supplemented scaffold was termed CHS-H-VEGF. *In vitro* and rabbit OA defect studies showed efficient loading and controlled release of VEGF, magnetic enhancement of osteogenic differentiation of MSCs, accelerated subchondral bone formation, and strong performance in osteochondral repair. The Fe_3_O_4_ component also enabled noninvasive MRI tracking; as microspheres were replaced by new bone, contrast signals changed, allowing real time monitoring of regeneration (Rong et al., 2024) (Figure 7E).

Hybrid exosomes, formed by fusing exosome and liposome membranes, combine convenient synthesis, high drug loading, biocompatibility, low immunogenicity, and potential tissue targeting (Mondal et al., 2023). Chen et al. developed HA based hydrogel microspheres carrying chondrocyte targeted hybrid exosomes that delivered a CRISPR/Cas9 system. Exosomes from HEK293 cells expressing CAP/EGFP-Lamp2b and Cas9 plasmids were fused with liposomes loaded with sgFGF18 to generate CAP/FGF18-hyEXO. *In vitro* and *in vivo*, this platform activated FGF18 and displayed chondrocyte targeting. Photopolymerization with HAMA produced CAP/FGF18-hyEXO@HMs microspheres. Under mechanical stimulation, the particles formed a self-renewing hydration layer that reduced the coefficient of friction and provided sustained lubrication. In an ACLT rat OA model, this system promoted chondrocyte proliferation, increased ECM synthesis, and supported matrix regeneration by regulating PI3K/AKT signaling; transcriptomics suggested synergy between *in vivo* FGF18 activation and lubrication (Chen M. et al., 2024) (Figure 7F). Using a similar microfluidic and photopolymerization strategy, Lei et al. prepared RAPA@Lipo@HMs, an HA based microsphere that released hydrogenated soy phosphatidylcholine liposomes under shear. The positively charged liposomes targeted the negatively charged cartilage surface to create a long-lasting boundary lubrication layer, while rapamycin removed excess ROS, limited chondrocyte apoptosis, and preserved homeostasis by enhancing autophagy (Lei et al., 2022).

Exogenous stimuli-responsive hydrogels enable on-demand gelation or drug release in joints in response to applied signals, thereby promoting cell migration and differentiation and facilitating matrix remodeling. Current use, however, is largely limited to animal studies and short-term validation, as translation is constrained by inadequate depth of stimulation and limited controllability in deep tissues. Design should match the stimulus modality to tissue accessibility depth and jointly tune energy dose, material response magnitude, and release cadence. This calibration can maximize lesion-site efficacy while minimizing exposure of surrounding tissues, and support procedural reproducibility and long-term safety.

## 5 Targeting mechanisms of aberrant neurovascular remodeling with smart responsive hydrogels

Aberrant interactions among chondral degeneration, subchondral sclerosis, pathologic angiogenesis, and neural remodeling reinforce one another and worsen osteoarthritis (OA). Abnormal remodeling in the subchondral compartment, together with neovessel formation and sensory fiber ingrowth, disrupts cartilage metabolic homeostasis and can directly or indirectly drive joint pain (Hu Y. et al., 2021). Subchondral bone lies between hyaline cartilage and the cement line and comprises the subchondral bone plate (SBP) and the underlying trabeculae. The SBP is richly vascularized and innervated, and trabeculae remodel continuously. Beyond providing mechanical support, this unit regulates solute exchange that influences cartilage nutrition and metabolism. In early OA, microstructural changes in the SBP limit exchange between bone and cartilage and disturb local metabolic balance (Pouran et al., 2017; Yang D. et al., 2024).

Hyaline cartilage is intrinsically antiangiogenic: factors such as chondromodulin-1, thrombospondin-1, and troponin I counter proangiogenic cues including VEGF, FGF, and TGF-β (Sun P. et al., 2020; Hu Y. et al., 2021). This barrier weakens in OA. Failure of protease inhibitor defenses permits vessels to cross the tidemark. OA chondrocytes show elevated pro- and anti-angiogenic mediators, yet inhibitor levels in deep cartilage remain near normal, favoring “bottom up” angiogenesis from the subchondral side (Fransès et al., 2010). Activated chondrocytes promote vascular invasion through mTORC1 signaling with partial upregulation of VEGF-A; enhanced capillary supply then further augments mTORC1 activity, creating a vicious cycle (Lu et al., 2018). Angiogenesis frequently coincides with axonal growth, as perivascular cells steer nascent axons by modulating guidance cues that shape OA pain pathways (Shalabi et al., 2024). Within the OA milieu, osteoblast-derived nerve growth factor (NGF) increases markedly, promoting both angiogenesis and nociception; NGF also induces FGF in a time- and dose-dependent manner, which enhances endothelial vascularization (Walsh et al., 2010; Yu et al., 2019). Multifunctional factors such as VEGF and TGF-β are highly expressed at the osteochondral junction and jointly drive neurovascular regrowth (Zhao L. et al., 2024). Clinically, newly formed nerve fibers accompany vessels that cross the tidemark into cartilage, and similar neurite ingrowth is observed in osteophytes (Suri et al., 2007). Because cartilage health depends on appropriate loading, perforations in the bone plate that contain new vessels and nerve fibers often colocalize with stress concentrations, linking biomechanical signals to plate remodeling and pain activation (Iijima et al., 2016). On this basis, smart hydrogels can be engineered to carry antiangiogenic or antineurotrophic agents for targeted release in the subchondral or intraarticular microenvironment, thereby modulating the neurovascular axis, slowing cartilage degeneration, and relieving pain. In parallel, hydrogels that mimic the three-dimensional architecture of the osteochondral junction may help reestablish normal neurovascular distribution and physiologic load transfer.

### 5.1 Smart hydrogels for osteoarthritis therapy by concurrently targeting the neurovascular axis

**FIGURE 8:**
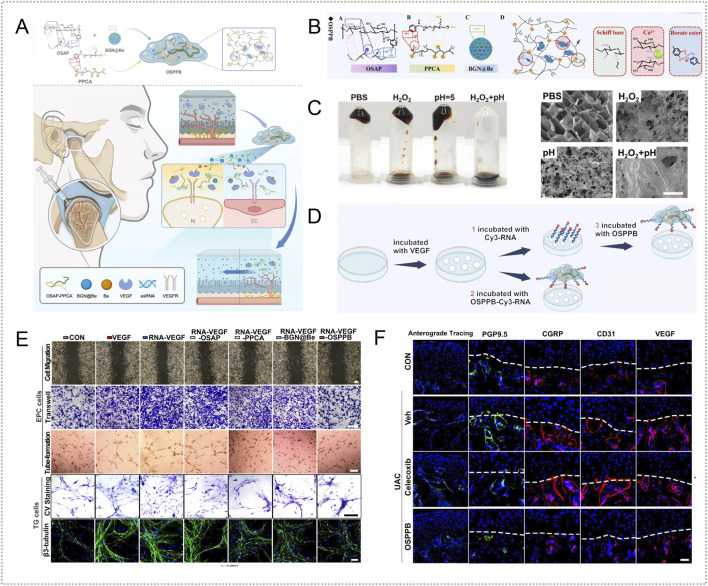
Targeting the Neurovascular Axis. **(A)** A dual responsive polycationic hydrogel (OSPPB) reverses temporomandibular joint osteoarthritis and alleviates chronic pain by suppressing neurovascularization at the osteochondral interface. **(B)** Network assembly: boronic acid groups on OSPPB form boronate esters with catechols on PPCA, while PPCA amines form Schiff base bonds with OSPPB aldehydes; bevacizumab-loaded bioactive glass nanoparticles (BGN@Be) are incorporated via Ca^2+^ coordination with OSPPB. **(C)** Under simulated low pH and high ROS, the hydrogel rapidly liquefies; combined stimulation reduces pore volume by 13.08%, indicating extensive dissolution of the network. **(D)** Mode of action: positively charged PPCA sequesters extracellular RNA, and bevacizumab in BGN@Be neutralizes VEGF activity. **(E)** *In vitro*, OSPPB inhibits angiogenesis and neurogenesis, markedly reducing the activity of trigeminal ganglion (TG) neuron like cells and endothelial progenitor cells (EPCs). **(F)** *In vivo*, OSPPB lowers levels of PGP 9.5, calcitonin gene-related peptide (CGRP), PECAM1/CD31 positive vessels, and VEGF, consistent with inhibition of neurovascularization and relief of persistent pain. Reproduced with permission from Ref. (Qin et al., 2025), Copyright 2025, Springer Nature.

In bone–vessel–cartilage crosstalk, chondrocyte mTORC1 upregulates VEGF-A, promotes H-type angiogenesis, and establishes positive feedback. Neutralization with the anti-VEGF-A antibody bevacizumab can interrupt this loop and inhibit neovessel formation (Lu et al., 2018; Pei et al., 2022). Extracellular RNA (exRNA) binds positively charged neurovascular factors and enhances their activity while contributing to inflammation, angiogenesis, and osteogenesis. It also mediates neurovascular remodeling in cardiovascular and joint disease by modulating VEGF signaling, among others (Preissner et al., 2020; Qin et al., 2023). Compared with blocking single effectors, clearing exRNA may offer a broader approach for OA pain. Qin and colleagues designed a hydrogel system, OSPPB (Figure 8A). Sodium alginate bearing aldehyde boronic-acid groups (OSAP) was crosslinked with a polyethyleneimine grafted protocatechuic-acid backbone (PPCA) through dynamic boronic-ester and Schiff-base bonds, and bevacizumab-loaded bioactive-glass nanoparticles (BGN@Be) were incorporated by coordination. Borate bonds formed between OSAP boronic acids and PPCA catechols, Schiff-base bonds formed between PPCA amines and OSAP aldehydes, and BGN coordinated with OSAP via Ca^2+^ (Figure 8B). In simulated low pH and high ROS conditions, the hydrogel rapidly liquefied; combined stimulation reduced pore volume by 13.08%, indicating substantial network disruption (Figure 8C). Positively charged PPCA sequestered and cleared exRNA, while BGN@Be neutralized VEGF activity (Figure 8D), thereby inhibiting both factor function and upstream recruitment and ultimately suppressing neurovascularization at the osteochondral interface. *In vitro*, the system markedly inhibited trigeminal-ganglion neuron-like cells and endothelial progenitor cells (Figure 8E). In unilateral anterior crossbite (UAC) mouse models of temporomandibular-joint OA, OSPPB produced analgesia comparable to celecoxib and improved pathology; levels of the neural marker PGP 9.5, calcitonin gene-related peptide (CGRP), PECAM1/CD31 positive vessels, and VEGF were significantly reduced (Figure 8F), indicating effective inhibition of neurovascularization, slower joint destruction, and relief of persistent pain (Qin et al., 2025).

### 5.2 Smart hydrogels for osteoarthritis therapy by inhibiting aberrant neurogenesis or angiogenesis

**FIGURE 9:**
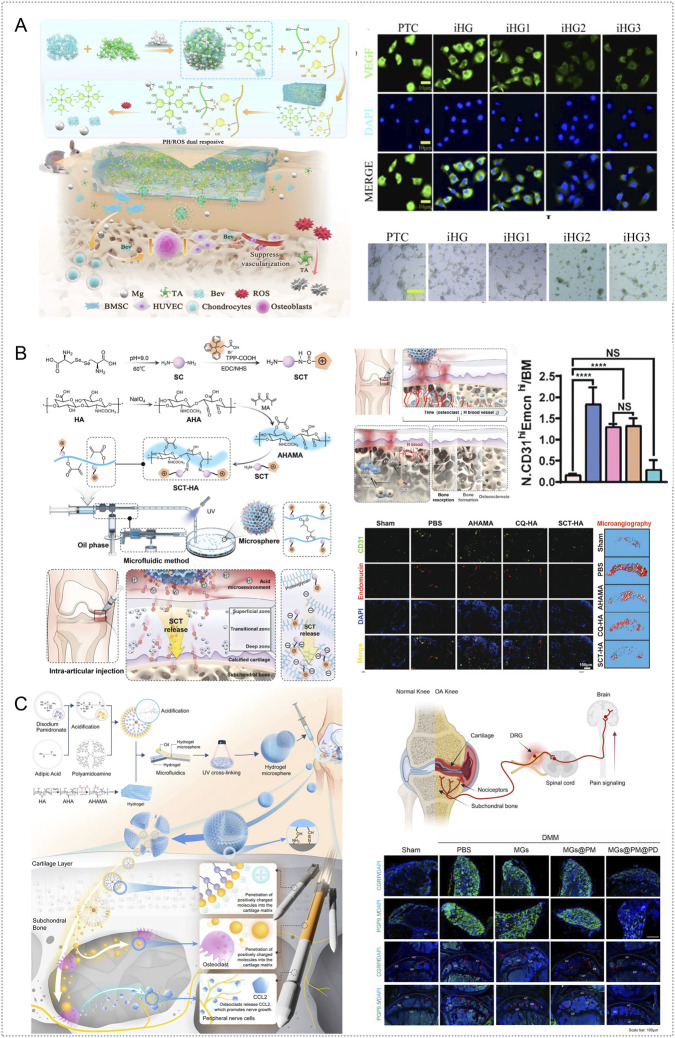
Inhibiting Aberrant Neurogenesis or Angiogenesis. **(A)** pH/ROS Dual-Responsive TA-Mg@Bev iHGs: TA-Mg@Bev is formed by loading bevacizumab onto a TA-Mg coordination network and embedding it into a hydrogel dynamically crosslinked with HA-PBA and PVA borate esters. In inflammatory microenvironments, this design enables controlled Bev release to suppress HUVEC angiogenesis and promote BMSC chondrogenic differentiation, thereby strengthening anti-vascular invasion. Reproduced with permission from Ref. (Chen H. et al., 2025), Copyright 2025, Elsevier. **(B)** pH-responsive SCT@AHAMA permeable microspheres: Selenium-doped carbon quantum dots (SCT) grafted with TPP are dynamically composited within an AHAMA matrix. Weakly acidic conditions trigger SCT release, allowing penetration into subchondral bone. SCT then scavenges mitochondrial ROS, inhibits osteoclast activation, and reduces H-type vascular (CD31^hi^Emcn^hi^) invasion, thereby blocking osteoclast-driven abnormal bone remodeling and the associated pathological vascular invasion. Reproduced with permission from Ref. (Zuo et al., 2024), Copyright 2024, Wiley-VCH GmbH. **(C)** Enzyme-responsive “carrier rocket” cascade delivery hydrogel microspheres (PD@PM@MG): AHAMA serves as the matrix and is loaded with PD@PM, a sixth-generation PAMAM nanocomposite modified with disodium pamidronate. Elevated hyaluronidase at the lesion site drives gradual microsphere degradation. Concurrent Schiff base formation enhances adhesion and retention, enabling sustained drug delivery to subchondral bone. This approach inhibits osteoclast-mediated bone resorption, lowers neuropathic markers (PGP9.5, CGRP), and alleviates pain. Reproduced with permission from Ref. (Zhu et al., 2025), Copyright 2025, Springer Nature.

Tannic acid (TA) is a natural polyphenol enriched in catechol and pyrogallol groups, with antioxidant and anti-inflammatory activity. It self-assembles with metal ions through coordination, π–π stacking, and hydrogen bonding to form metal-polyphenol networks (Zhao et al., 2024c). Mg^2+^, an essential physiological cation, modulates local inflammation and supports angiogenesis, yet its mild coordination chemistry rarely denatures proteins (Wang X. et al., 2025). After cartilage injury, the loss of an anti-vascular invasion barrier permits neovessels from subchondral bone to infiltrate normally avascular cartilage, promoting ossification and functional decline; the accompanying rise in oxygen tension further disrupts the intrinsic hypoxic microenvironment (Fransès et al., 2010). Against this background, Chen et al. developed a pH/ROS dual-responsive hydrogel composite to enhance anti-angiogenic efficacy and accelerate cartilage repair. The core comprises bevacizumab (Bev)-loaded trimeric association nanopolymers (TA-Mg@Bev), formed via phenolic hydroxyl-mediated hydrogen bonding and polyphenol-metal coordination that incorporate Bev and Mg^2+^. TA-Mg@Bev is embedded within a hydrogel network dynamically crosslinked by borate bonds between HA-PBA and PVA, yielding TA-Mg@Bev iHGs. Reversible borate interactions impart stimulus responsiveness and reinforce mechanics through additional phenolic hydroxyl-mediated hydrogen bonding, enabling sustained Bev release in inflammatory microenvironments. Through Bev-mediated VEGF blockade, the system suppresses HUVEC proliferation and angiogenic behavior, while promoting BMSC proliferation and chondrogenic differentiation and limiting chondrocyte hypertrophy (Chen H. et al., 2025) (Figure 9A). Furthermore, because activated vascular endothelial cells (CD62E^+^) drive aberrant angiogenesis and amplify inflammation in OA, researchers developed a CD62E/ROS dual-responsive ETS/CS microsphere. Using silk fibroin as the carrier, the E-selectin binding peptide (ESBP) enables targeted enrichment toward CD62E^+^ activated vascular endothelial cells, whereas the thioketal (TK) structure triggers ROS-responsive release of clematis triterpenoid saponins (CS). CS restrains endothelial activation and angiogenesis by restoring mitochondrial homeostasis via OPA1 and dampens the endothelial-macrophage inflammatory cascade. *In vivo* cartilage defect models further show reduced angiogenesis and inflammation, together with enhanced cartilage repair (Tu et al., 2024).

Abnormal remodeling of subchondral bone (SB) accelerates cartilage destruction and osteoarthritis (OA), making restoration of cartilage–bone metabolic homeostasis a central therapeutic goal. Osteoclasts contribute not only by releasing growth factors that disrupt chondrocyte metabolism, but also by promoting H-type vascularization and abnormal nerve fiber formation. These changes shift the SB niche from resorption toward sclerosis, with neurovascular invasion into cartilage and inflammatory infiltration that further destabilize cartilage phenotype and metabolic balance (Su et al., 2020). Yet most therapeutics penetrate cartilage poorly and fail to reach SB at effective levels. To overcome this barrier, Zuo et al. exploited the ability of quantum dots to traverse cartilage and access SB. Ultra-small selenium-doped carbon quantum dots (Se-CQD, SC) were conjugated with TPP to form SCT, which was then dynamically composited with AHAMA to generate highly permeable micro/nanoscale hydrogel microspheres (SCT@AHAMA). In the weakly acidic OA microenvironment, Schiff base bonds cleave to enable sustained release of ultrafine SCT. The released SCT overcomes cartilage steric hindrance, penetrates SB, and helps rebalance cartilage matrix synthesis and catabolism. *In vitro*, selenium atoms scavenge ROS in macrophage mitochondria, inhibit osteoclast differentiation and function, and suppress early chondrocyte apoptosis to maintain equilibrium between cartilage matrix synthesis and degradation. *In vivo*, the system suppresses osteoclastogenesis and H vascular invasion, thereby modulating the initiation and progression of aberrant bone remodeling and limiting SB-associated cartilage degeneration (Zuo et al., 2024) (Figure 9B). Concurrently, Zhu et al. proposed a “carrier rocket” cascade delivery hydrogel microsphere system that uses AHAMA hydrogel microspheres as an intra-articular drug reservoir loaded with pamidronate disodium-modified sixth-generation PAMAM nanocomposites (PD@PM). Elevated hyaluronidase at the lesion site drives gradual microsphere degradation and release of PD@PM. Meanwhile, aldehyde groups form Schiff bases with tissue amines, enhancing adhesion and retention to support sustained delivery to subchondral bone and inhibit osteoclast differentiation and bone resorption. *In vitro*, this system reduces osteoclast differentiation and resorptive activity and improves inflammation-related metabolic imbalance in the chondrocyte matrix. *In vivo*, it significantly alleviates pain-related behavioral manifestations, decreases sensory nerve-related markers such as PGP9.5 and CGRP, suppresses abnormal SB remodeling, and mitigates cartilage degeneration (Zhu et al., 2025) (Figure 9C).

Current efforts to directly target neural remodeling in osteoarthritis remain relatively limited. Most strategies instead modulate pain transmission and neuroinflammation through neural pathways, producing analgesia and disease mitigation via exogenous stimulation or targeted delivery. For example, Li et al. developed ultrasound-responsive piezoelectric analgesic microspheres that generate electrical pulses at dorsal root ganglion (DRG) nerve endings under ultrasound irradiation. By promoting slow inactivation of the voltage-gated sodium channel Na_v_1.7, these microspheres reduce DRG excitability, block nociceptive signaling, and improve osteoarthritis-associated pain behavior (Li et al., 2025d). In contrast, Liu et al. developed an injectable thermosensitive hydrogel, KAF@PLEL, that enables sustained delivery of anti-inflammatory peptides to the DRG. By reprogramming DRG macrophages from a pro-inflammatory to an anti-inflammatory phenotype, KAF@PLEL suppresses neuroinflammation and provides durable relief of chronic osteoarthritis pain (Liu Y. et al., 2025). Looking ahead, smart hydrogel therapies for osteoarthritis should prioritize abnormal nerve fiber invasion at the cartilage–bone interface and neural sensitization, using controlled-release designs to inhibit neural remodeling. This framework could relieve persistent pain at its source while synergistically slowing joint degeneration.

In sum, aberrant vascular and neural remodeling is central to OA progression and chronic pain. Neurovascularization at the subchondral bone–cartilage interface not only initiates early pathology but also fuels inflammation, tissue damage, and maintenance of nociceptive signaling. Targeting this axis offers a precision strategy that can modulate disease activity while managing pain. Work on smart hydrogels for directed control of neurovascular remodeling in OA is still at an early stage, and coordinated designs for simultaneous vascular–neural regulation remains limited. Future platforms may integrate anti-NGF agents, endothelial-targeted aptamers, nerve fiber blocking peptides, and exosome carriers to build next-generation interventions that address the vascular–nerve–pain axis and jointly optimize structural repair and analgesia.

## 6 Conclusion and future perspective

Current therapies for osteoarthritis (OA) largely aim to relieve pain and slow cartilage loss, yet precise multitarget intervention within the complex OA microenvironment remains inadequate. Conventional hydrogels for osteoarthritis have largely focused on integrating components of the regenerative microenvironment, including mesenchymal stem cells and molecules that mediate adhesion or lubrication, to promote repair; however, they have not systematically adopted stimuli-responsive behavior or multitarget regulatory strategies (Kalairaj et al., 2024). Smart responsive hydrogels have therefore emerged as a promising direction in OA biomaterials research (Berenbaum, 2013). This review has summarized advances in their use for anti-inflammatory control, mitochondrial modulation, cartilage regeneration, and correction of aberrant neurovascular remodeling, underscoring their potential to build microenvironment-adaptive therapeutic platforms. Over recent decades, hydrophilic biomaterials have attracted broad interest and shown multiple advantages in biosensing, disease treatment, and especially wound dressings (Correa et al., 2021). By contrast, truly approved hydrogel products for OA remain scarce. A search of ClinicalTrials.gov indicates that most ongoing hydrogel studies for OA still evaluate conventional systems; for example, a randomized, multicenter trial (NCT04045431) assessed polyacrylamide hydrogel in knee OA (Bliddal et al., 2023). While translation of traditional hydrogels is progressing, smart hydrogels face hurdles that include more complex synthesis and functionalization, cumbersome preparation, variable batch quality, and higher costs, all of which impede adoption. Moreover, most studies remain confined to animal models with joint anatomy and physiology that differ from humans; systematic mechanistic work and long-term clinical follow-up are limited, preventing standardization of treatment strategies. In addition, many smart hydrogels are regulated as combination products that integrate a device-like scaffold with drug, biologic, cell, or gene payloads. This raises pathway-defining issues such as primary mode of action, cross-component quality systems, and aligned CMC evidence for safety, performance, and clinical benefit. Advancing smart hydrogels from laboratory concepts to clinical application is therefore a key priority for precision therapy and tissue reconstruction in OA.

Several areas merit focus to accelerate clinical readiness. First, mechanism-driven, multitarget, time-controlled regulation should be strengthened. Most current materials emphasize inflammation control and cartilage repair, yet OA pathogenesis is dynamic. Future systems should integrate anti-inflammatory functions, maintenance of mitochondrial homeostasis, chondrogenic promotion, and neurovascular regulation into a single platform that is responsive to both endogenous and exogenous cues. Such designs would enable on-demand, stepwise, and region-specific release while balancing lubrication with adhesion to improve residence time and local bioavailability. Second, targeted mitochondrial intervention should be advanced. Existing approaches rely mainly on endogenous triggers within lesions. Combining endogenous and exogenous activation with mitochondria-targeting ligands could support on-demand delivery of antioxidants, NAD^+^ metabolism enhancers, and nucleic acid therapeutics that induce autophagy or prevent chondrocyte death. In parallel, standardization of readouts including mitochondrial membrane potential, ATP content, respiratory phenotypes, mitochondrial DNA damage, and markers of the mitochondrial unfolded protein response will be essential for long term safety assessment. Third, remodeling the neurovascular axis is needed to balance analgesia with structural repair. By focusing on subchondral angiogenesis and nociceptive fiber ingrowth, hydrogels that deliver anti-VEGF or NGF inhibitors could shift treatment from simple anti-inflammatory analgesia toward structural and functional reconstruction. At the materials level, lubrication must be matched with mechanical compatibility; where appropriate, piezoelectric or triboelectric units could convert joint motion into micro-electrical stimulation to relieve pain while limiting matrix degradation and abnormal bone remodeling. Finally, systematic work on clinical applicability and translational evaluation is critical. Priorities include mild, rapid, and efficient gelation strategies (bioorthogonal reactions, shear-injectable formulations, self-healing, and reversible dynamic crosslinking); careful balancing of self-healing efficiency with load-bearing mechanics; and attention to cellular safety for chemistries such as slow Schiff-base formation and thiol radical reactions. For multi-component systems, batch-to-batch consistency must be demonstrated beyond bulk mechanics and gelation, covering cargo content, release kinetics, and microstructure (including nanoparticle or MOF dispersion and any spatial gradients) with validated assays and predefined specifications. Equally important, degradation should be evaluated as a long-term exposure scenario, including local leachables, biodistribution, and chronic joint safety. This is particularly relevant for metal-containing platforms (for example, ZIF-8), where ion release during degradation may perturb ionic homeostasis and inflammatory signaling, necessitating rigorous extractables and extended biocompatibility testing. Procedures for arthroscopic or ultrasound-guided delivery and *in situ* fixation should be refined, with prevalidation of sterilization compatibility, batch consistency, and scalable manufacturing. Disease models, imaging and tracking protocols, and functional endpoints should be defined to enable multicenter studies with long term follow-up, thereby establishing reproducible, regulatory-compliant, and scalable pathways.

Overall, smart responsive hydrogels provide a robust foundation for future precision interventions in OA. By integrating materials science with disease-mechanism research and translational medicine, the field may progress from symptomatic management to comprehensive structural repair and functional restoration, offering new prospects for integrated OA care.
